# A 5′ UTR GGN repeat controls localisation and translation of a potassium leak channel mRNA through G-quadruplex formation

**DOI:** 10.1093/nar/gkaa699

**Published:** 2020-09-01

**Authors:** Connor J Maltby, James P R Schofield, Steven D Houghton, Ita O’Kelly, Mariana Vargas-Caballero, Katrin Deinhardt, Mark J Coldwell

**Affiliations:** School of Biological Sciences, University of Southampton, Southampton, Hampshire SO17 1BJ, UK; School of Biological Sciences, University of Southampton, Southampton, Hampshire SO17 1BJ, UK; School of Biological Sciences, University of Southampton, Southampton, Hampshire SO17 1BJ, UK; Centre for Human Development, Stem Cells and Regeneration, University of Southampton, Southampton, Hampshire SO17 1BJ, UK; School of Biological Sciences, University of Southampton, Southampton, Hampshire SO17 1BJ, UK; School of Biological Sciences, University of Southampton, Southampton, Hampshire SO17 1BJ, UK; School of Biological Sciences, University of Southampton, Southampton, Hampshire SO17 1BJ, UK

## Abstract

RNA G-quadruplexes (G4s) are secondary structures proposed to function as regulators of post-transcriptional mRNA localisation and translation. G4s within some neuronal mRNAs are known to control distal localisation and local translation, contributing to distinct local proteomes that facilitate the synaptic remodelling attributed to normal cellular function. In this study, we characterise the G4 formation of a (GGN)_13_ repeat found within the 5′ UTR of the potassium 2-pore domain leak channel Task3 mRNA. Biophysical analyses show that this (GGN)_13_ repeat forms a parallel G4 *in vitro* exhibiting the stereotypical potassium specificity of G4s, remaining thermostable under physiological ionic conditions. Through mouse brain tissue G4-RNA immunoprecipitation, we further confirm that Task3 mRNA forms a G4 structure *in vivo*. The G4 is inhibitory to translation of Task3 *in vitro* and is overcome through activity of a G4-specific helicase DHX36, increasing K^+^ leak currents and membrane hyperpolarisation in HEK293 cells. Further, we observe that this G4 is fundamental to ensuring delivery of Task3 mRNA to distal primary cortical neurites. It has been shown that aberrant Task3 expression correlates with neuronal dysfunction, we therefore posit that this G4 is important in regulated local expression of Task3 leak channels that maintain K^+^ leak within neurons.

## INTRODUCTION

DNA and RNA can adopt many secondary structures that have important roles in dictating cellular processes. G-quadruplexes (G4s) form in portions of guanine-rich DNA and RNA, and are amongst the most stable of nucleic acid secondary structures (1). G4s are more stable in RNA than DNA and can exist as mono- or multi-stranded structures (2). G4s comprise stacks of guanine tetrads that stabilise via non-canonical Hoogsteen hydrogen bonding, as opposed to the canonical Watson-Crick bonding observed in duplex DNA/RNA. Stacked G-tetrads are stabilised through interactions with monovalent cations, where K^+^ elicits the greatest stabilisation, whereas Li^+^ is unable to stabilise G4 formation due to constraints in ionic radii within the G4 central electronegative pore (3,4).

G4s are enriched within regulatory elements of both genomic DNA and mRNAs (5,6), clustering at telomeres (7), promoter regions and transcription start sites of genomic DNA (8–10), as well as splice sites (11) and untranslated regions (UTRs) of mRNAs (12–14). G4s in mRNAs are thought to be of particular importance in the regulation of gene expression, with G4s within the 5′ UTR of mRNAs able to inhibit translation by impeding scanning of the 40S ribosomal subunit, or promoting translation through non-canonical ribosomal interactions with internal ribosomal entry sites (IRES) (15). Similarly, G4s within 3′ UTRs can impede translation through association with regulatory proteins (16), or indirectly through miRNA recruitment (17) as well as regulating polyadenylation (18) and subcellular localisation of mRNAs (19–21).

Local translation is of particular importance within highly polarised cells, such as within neurons where singular cortical axons can project contra-laterally across the diameter of the brain. As such, both protein and mRNA transport to these distal sites of neurons is critical to neuronal homeostatic functions (22). Recently, nearly 2000 actively translating mRNAs were identified within the axon through an axon-specific translating ribosome affinity purification (TRAP) approach (23,24). Similarly, identification of translating polyribosomes and over 2500 distinct mRNA transcripts through deep-sequencing within the hippocampal neuropil (25,26) further suggests that neurons have a dependency on the local translation of mRNAs in distal neurite compartments (22,27).

Disruption of G4 homeostasis has been implicated in several neurodevelopmental and degenerative diseases. A G4-forming (CGG)_n_ repeat expansion within mutant forms of the *FMR1* gene locus induces aberrant expression of the G4 binding protein FMRP which leads to the neurodevelopmental Fragile-X syndrome (FXS), or the repeat-length-dependent neurodegenerative Fragile-X tremor and ataxia syndrome (FXTAS) (28,29). Similarly, the G4-forming hexanucleotide (GGGGCC)_*n*_ repeat expansion within the spliced intron 1 of C9Orf72 causes the most common form of inherited amyotrophic lateral sclerosis (ALS), and/or frontotemporal dementia (FTD), through RNA toxicity and repeat-associated non-AUG (RAN) translation producing toxic dipeptide-repeat species (30). As such, the importance of G4s during development and maintenance of synaptic connections through regulated mRNA localisation, as well as their role during neuronal dysfunction and neurodegeneration (28,31) is of particular interest.

**Figure 1.**
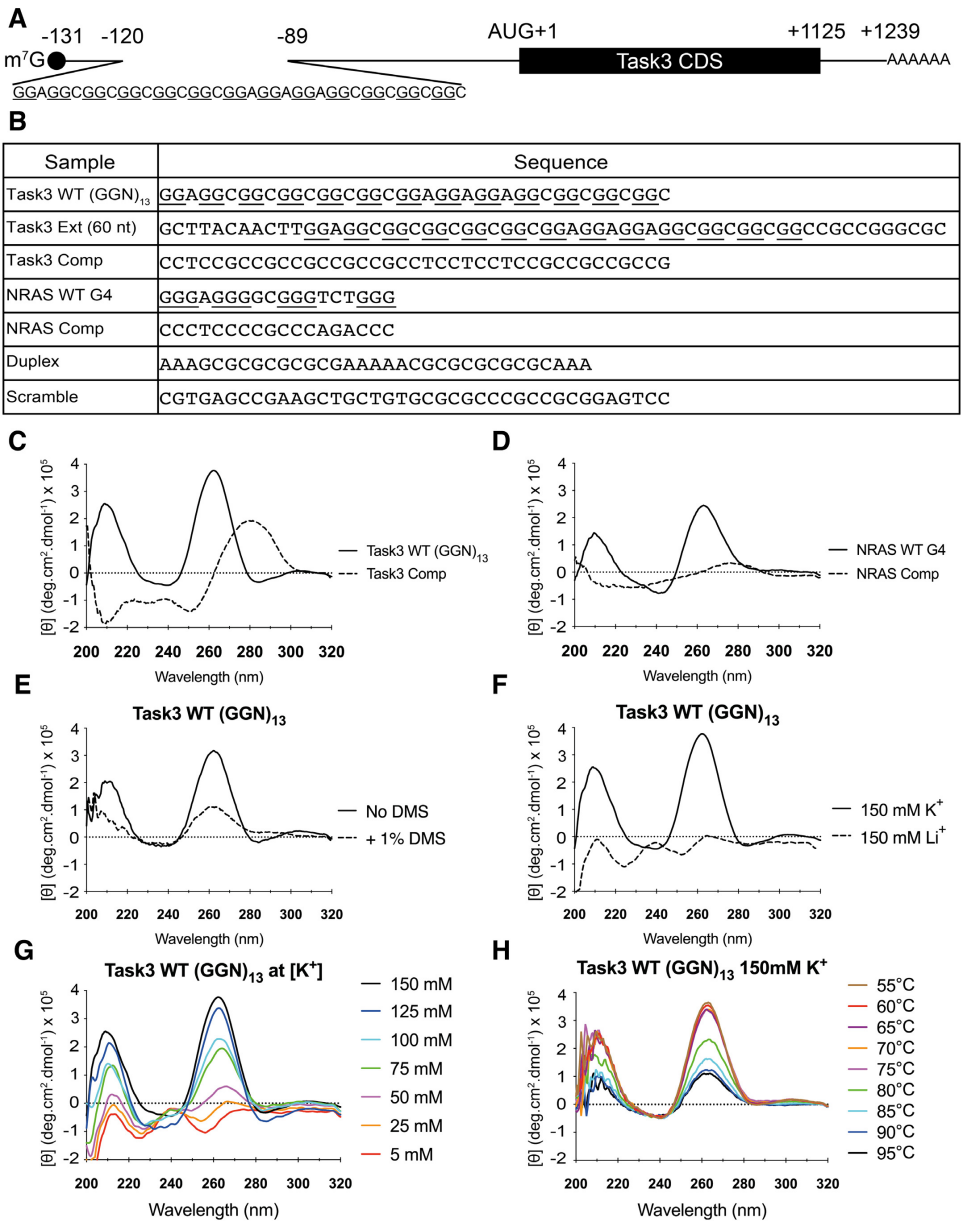
Task3 mRNA contains a 5′ UTR (GGN)_13_ repeat that forms a parallel G4 *in vitro*. (**A**) Schematic of Task3 mRNA indicating the (GGN)_13_ repeat within the 5′ UTR, as identified by 5′ RACE (GenBank: MN510330). (**B**) Sequences of control and experimental oligonucleotides used for biophysical analyses. (**C**, **D**) The CD spectra of 10 μM Task3 WT (GGN)_13_ and Task3 Complementary oligos (C) or NRAS WT G4 and NRAS complementary sequence oligonucleotides (D) folded in potassium phosphate buffer supplemented with KCl to 150 mM K^+^. (**E**) The CD spectra of 10 μM Task3 WT (GGN)_13_ oligos folded with or without pre-treatment of 1% dimethyl sulphate (DMS) to induce N7 methylation of guanine and occlude G-quartet formation. (**F**) The CD spectra of 10 μM Task3 WT (GGN)_13_ oligonucleotides folded in potassium phosphate buffer supplemented with KCl to 150 mM K^+^ to facilitate G4 formation (CD spectra overlaid from 1C) or folded in sodium phosphate buffer supplemented with LiCl to 150 mM Li^+^ to inhibit G4 formation. (**G**) The CD spectra of 10 μM Task3 WT (GGN)_13_ oligonucleotides folded in potassium phosphate buffer supplemented with KCl to a free [K^+^] of between 5 and 150 mM K^+^. (**H**) The CD thermal profile of 10 μM Task3 WT (GGN)_13_ oligonucleotides between 55 and 95°C, data omitted <55°C. *N* = 3 experimental replicates of five repeated acquisitions for signal:noise correction with representative traces shown for each condition.

The TWIK-related acid-sensitive K^+^ channel (Task3, also known as K2P9.1) is a potassium two-pore domain (K2P) leak channel responsible for regulating the resting membrane potentials of various cell types (32). Task3 is enriched in brain regions associated with high rates of neuronal firing such as the hippocampus and cerebellum (33), and is required during processes such as synaptic plasticity. Task3 loss of function leads to the maternally-imprinted intellectual disability disorder Birk-Barel mental retardation (34), and perturbations in Task3 expression lead to changes in resting membrane potentials and neuronal action potential kinetics and recovery (33), suggesting that tight regulation of expression is essential. In this study, we analyse a novel (GGN)_13_ repeat in the cap-proximal region of the 5′ UTR of Task3 mRNA (Figure 1A) that folds into a parallel G4 structure both *in vitro* and *vivo* responsible for the regulation of distal Task3 mRNA localisation and translational regulation required for the maintenance of normal cellular physiology.

## MATERIALS AND METHODS

### Materials

Primary antibodies used in this study were anti-FLAG M2 (Merck, F1804), anti-α-Actin (Sigma, AC-40), anti-Task3 (Almone, APC-044), anti-DHX36 (Proteintech, 13159), anti-α-Tubulin (Proteintech, 66031), anti-Lamin A+C (Cell Signalling Technology, 2032), BG4 (Absolute, Ab00174-24.1). Secondary antibodies used were conjugated to Alexa Fluor^®^ fluorophores (Invitrogen) or IRDyes (LI-COR Biosciences). The plasmids used were cloned from human brain cDNA (Agilent 540005-41) into C-terminal pcDNA3.1-3F/eGFP vectors as previously described (35), and mutants created through site-directed mutagenesis (Promega). Primers were obtained from Sigma, with sequences outlined in Supplementary Figure S6. Stellaris^®^ RNA FISH probes were obtained from LGC Biosearch Tech. Anti-Task3 mouse RNA FISH probes were designed using the Stellaris^®^ online web tool (see data availability) and conjugated to Quasar^®^ 570 nm fluorophores. Anti-eGFP RNA FISH probes were pre-designed from Stellaris^®^ and conjugated to Cal Fluor^®^ Red 590 nm fluorophores.

### Circular dichroism spectroscopy

Oligonucleotides were dissolved in nuclease-free H_2_O to a concentration of 1 mM stock solution and diluted to an experimental concentration of 10 μM, estimated through OD_260_ absorption, in buffer containing potassium or sodium phosphate and potassium/lithium chloride salt to the desired free [K^+^/Li^+^]. The oligonucleotide preparations were then folded by heating to 95°C for 10 min and cooled at a rate of 0.5°C/min to room temperature (RT).

CD spectra were recorded using a Jasco J-720 spectropolarimeter equipped with a Jasco PS-450 xenon lamp and a Grant LTD6G temperature-controlled cell holder. Quartz cuvettes of 1 mm path length were used, and scans were recorded at a scan speed of 50 nm/min with response time of 2 s. The wavelength of scans was recorded between 320 and 200 nm, with 0.5 nm resolution and sensitivity of 20 m°. The average of five spectral scans was taken for each sample followed by baseline buffer correction. All scans were conducted at 25°C except where temperature is the designated independent variable. The data were normalised to molar ellipticity using the equation: [θ] = (100 × θ)/(*c* × *l*), where *c* = molar concentration and *l* = pathlength in cm. Temperature controlled experiments were achieved through water-conductive heating of the cell holder. Temperatures were increased from 20°C to 95°C at 5°C increments, allowing 5 minutes stabilisation at each temperature before spectra were obtained. All samples were overlaid with mineral oil to prevent evaporation.

### Cell culture

HEK293 cells were maintained in DMEM (Dulbecco's Modified Eagle's Medium) media (Gibco) with 10% FBS supplement (Gibco) and incubated at 37°C and 5% CO_2_. Cells were transfected using Genejuice™ (Novagen) as described (36) and proteins or mRNAs extracted after 48 h.

For tissue collection, C57Bl/6 wildtype mice were sacrificed in accordance with the Animals (Scientific Procedures) Act 1986 as approved by the UK Home Office. Primary neurons were prepared in Dulbecco's PBS (DPBS, Life Technologies) from cortices of E15–E18 mice. Neurons were dissociated and maintained in complete NBM (Neurobasal medium with 2% (v/v) B27, 0.5 mM Glutamax) as previously described (37). The number of cells was determined and plated prior to transfection. For immunocytochemistry/RNA FISH, cells were transfected on day in vitro (DIV) 7 with Lipofectamine 2000 as described (38). The lipoplex mixture was added to the cells and incubated at 37°C for 40 min. Subsequently, the lipoplex-containing medium was removed from the wells and replaced with conditioned medium. For bicuculline treatment, the cells were treated with 50 μM (+)-bicuculline (Tocris) or 0.1% DMSO (vehicle treatment) prepared in complete NBM. Cells were incubated at 37°C for 24, 12, 6, 3, 1 h and 15 min and then harvested. For glutamate treatment, the cells were treated with 50 μM L-glutamic acid, monosodium salt monohydrate (Sigma) dissolved in ddH_2_O prepared in complete NBM conjunction with a media change control. Cells were incubated for 30, 60 or 120 s, media changed and then allowed to recover for 1 h at 37°C before being harvested.

For protein/RNA isolation using Corning^®^ Costar^®^ Transwell^®^ inserts (24 mm diameter, 3491), murine embryonic cortical neurons were seeded at the desired density and protein/RNA harvested at DIV10. Axonal preparations were isolated as described (39).

### HEK293 cell recordings

HEK293 cells were maintained and transfected as above and whole-cell patch clamp was performed after 24 h transfection. Thick-glass borosilicate glass micropipettes were pulled to a tip resistance of 5–7 MΩ then filled with intracellular recording solution containing: 115 mM potassium gluconate, 10 mM KCl, 10 mM HEPES, 10 mM potassium phosphocreatine, 0.4 mM GTP, 4 mM ATP, and pH balanced to 7.3 with KOH, osmolarity of ∼280 mOsm. Cells were perfused with artificial cerebrospinal fluid (ACSF) kept at 37°C throughout recording. ACSF contained: 126 mM NaCl, 2 mM CaCl_2_, 10 mM glucose, 2 mM MgSO_4_·7H_2_O, 3 mM KCl, 1.25 mM NaH_2_PO4.2H_2_O and 26.4 mM NaHCO_3_, bubbled with carbogen gas (95% O_2_, 5% CO_2_), osmolarity of ∼ 300 mOsm. The membrane potential was measured using a Multiclamp 700B (Molecular Devices) in current clamp (bridge) mode. Membrane potential was corrected for liquid junction potential of −12.5 mV which was measured directly (40).

### Western blotting

Proteins were extracted in Radioimmunoprecipitation assay (RIPA) buffer (150 mM NaCl, 1% (v/v) NP-40, 1% (w/v) DOC, 0.1% (v/v) SDS, 50 mM Tris–HCl pH 7.6, 1 mM EDTA, 1 mM EGTA, 1X Halt protease and phosphatase inhibitor cocktail (Thermo)) as previously described (41). Protein lysates were prepared and blotted following standard procedures, and images were captured using the Odyssey IR Imaging System (LI-COR Biosciences). Image Studio Scanner software was used to obtain the image, and Image Studio Lite software was used to quantify the intensities of the bands.

### RNA Immunoprecipitation (RIP)

For mouse brain RNA IP, adult C57Bl/6 wildtype mice were sacrificed as described above and brains isolated on ice. Meninges were removed and cerebral cortices separated with half a cerebral cortex homogenised in 500 μl ice-cold G4RP buffer (42) (150 mM KCl, 25 mM Tris–HCl pH7.4, 5 mM EDTA, 0.5 mM DTT, 0.5% NP40, RNase inhibitor (Promega), 1× Halt Protease Inhibitor Cocktail (Thermo Scientific)) using a mechanical homogeniser. For transfected RNA IPs, HEK293 cells were seeded at a density of 10^6^ in 10 cm dish, transfected as outlined above and resuspended in 500 μl ice-cold G4RP buffer. Homogenates were then sonicated on ice using a Fisherbrand Model 705 Sonic Dismembrator with 3 mm microtip probe at 10% duty for 10 s followed by 30 s rest, repeated 12 times for 2 min total sonication. 5% homogenate was removed and snap frozen as an input control, after which 3 μg antibody (materials) was added to the remaining homogenate and incubated at 4°C overnight on a rotator. 20 μl protein A/G beads (Santa Cruz, sc-2003) were subsequently added with a wide-bore pipette tip and incubated at 4°C for 2 h on a rotator. Homogenates were then centrifuged for 5 min 2000 × g at 4°C and supernatant collected as flow-through control. Beads were subsequently washed 3× in 500 μl ice-cold G4RP buffer before being incubated at 70°C for 1 h to release bound antibody and RNAs. RNAs from input, IP and flow fractions were then isolated through TRIzol (Invitrogen) extraction.

### RT-PCR and RT-qPCR

RNAs were extracted using the NucleoSpin^®^ RNA extraction kit (Machery Nagel) according to manufacturer's instructions. RNAs were reverse transcribed to cDNA using the qScript cDNA Supermix (QuantaBio) according to manufacturer's instructions. Endpoint PCRs were carried out using Readymix™ Taq PCR Reaction Mix (Sigma) with primers obtained from Sigma (see materials and Supplementary Figure S6). Quantitative PCR (qPCR) amplification was performed using SYBR^®^ Select Master Mix (Life Technologies) and an Eco qPCR System (Illumina) using the following conditions: initial denaturation 95°C for 5 min, 40 cycles of denaturation at 95°C for 10 s, and data collection at 60°C for 1 min. All reactions were performed with two technical replicates. The expression level was normalised to the level of the reference genes stated and quantified using the ΔΔCt method.

### RNA Fluorescence *in Situ* Hybridisation (FISH) and Fluorescent Immunocytochemistry (ICC)

At the desired timepoint, cells were washed in ice-cold DPBS and fixed in 4% (v/v) paraformaldehyde. For RNA FISH, cells were permeabilised using DPBS containing 0.1% (v/v) Triton X-100 and washed with Stellaris^®^ Wash Buffer A (with 10% (v/v) formamide). Fluorescent probes were then diluted into hybridisation buffer (30 mM saline sodium citrate buffer, 0.2% (w/v) BSA, 10% (w/v) dextran sulphate, 50% (w/v) formamide) to a concentration of 125 nM and 100 μl was placed onto coverslips. Coverslips were incubated in a light-protected humidified chamber at 37°C for 16 h. Coverslips were transferred to 1 ml Stellaris^®^ Wash Buffer A containing 375 nM Hoechst stain (Sigma) and incubated in a light-protected humidified chamber at 37°C for 30 min. Coverslips were then washed in Stellaris^®^ Wash Buffer B and mounted for imaging. For ICC, cells were permeabilised using DPBS containing 0.1% (v/v) Triton X-100, washed with TBS and blocked in 10% (v/v) normal goat serum (NGS) in TBS. Primary antibodies were subsequently diluted into 10% (v/v) NGS and overlaid onto coverslips for 1 h at RT. Coverslips were further washed and overlaid with 10% (v/v) NGS with the respective secondary antibody and incubated in a light-protected container for 30 min at RT. Coverslips were finally washed in containing 375 nM Hoechst stain (Sigma) and incubated in a light-protected humidified chamber at 37°C for 30 min before mounting.

Fluorescent preparations were imaged using a ×60/1.42 NA Oil Plan Apo objective on a DeltaVision Elite system (GE Life Sciences) with an SSI 7-band light-emitting diode for illumination and a monochrome sCMOS camera using SoftWoRks software (version 6). 4′,6-Diamidino-2-phenylindole, FITC, TRITC, Cy-5 and DIC channels were used. The images were analysed using the Fiji image processing package (NIH).

## RESULTS

### Task3 (GGN)_13_ forms a parallel G-quadruplex *in vitro* under physiological [K^+^]

5′ Rapid amplification of cDNA ends (5′ RACE) sequencing of full-length Task3 5′ UTR from human brain mRNA revealed a 71 nucleotide (nt) 5′ UTR N-terminal extension, which when sequenced was found to contain a (GGN)_13_ repeat 11 nt downstream of the m^7^G 5′-cap (Figure 1A). GGN repeats can form G4s as well as intermittently bulged duplex secondary structures within mRNA (43). To investigate the structural properties of this 5′ UTR (GGN)_13_ repeat *in vitro*, we used circular dichroism spectroscopy (CD) using DNA oligonucleotides of various sequences of interest (Figure 1B). At a physiological concentration of 150 mM K^+^, Task3 wild-type (WT) (GGN)_13_ forms a parallel G4, exhibiting the spectral maxima (λ_max_) of 210 and 261 nm (Figure 1C) previously reported for and consistent with parallel G4 formation (44,45), which was also observed for NRAS WT G4 (Figure 1D). DNA oligonucleotide spectra were validated against bona fide RNA oligonucleotide spectra for Task3 (GGN)_13_ showing overlapping traces (Supplementary Figure S1A) and conserved ion selectivity (Supplementary Figure S1B), thus confirming previously reported observations that DNA analogues of parallel RNA G4s show the same G4 forming ability and CD traces (45).

To further test G4 formation for Task3 (GGN)_13_, we compared the CD spectra of the WT G4 forming sequence to the complementary (CCN)_13_ sequence to prevent G4 formation without disrupting the ability for any potential duplex formation through canonical Watson-Crick base pairing by maintaining GC base composition. At 150 mM K^+^, the Task3 complementary (CCN)_13_ sequence exhibits a λ_max_ at ∼280 nm and λ_min_ at 210 and 250 nm (Figure 1C), suggesting formation of either a B-form duplex or intercalated motif (i-motif) (46,47), and so the formation of a different, non-G4 structural conformation when compared to Task3 WT (GGN)_13_. From this, it is likely the presence of doublet guanine-tracts that is contributing to the structural conformation observed for Task3 WT (GGN)_13_, and not Watson-Crick base pairing due to the distinct non-B conformation. However, a loss of any defining λ_max/min_ is observed when complementing the NRAS WT G4 sequence, suggesting that no structure is likely to be forming (Figure 1D). Further, no defining structural characteristics were observed through CD for a highly GC-rich scrambled sequence (Supplementary Figure S1C), further suggesting topological arrangement of GC bases is more important for structural formation than generic GC-base composition.

Hoogsteen hydrogen bonding is fundamental to G4 formation. We therefore sought to inhibit G4 formation through pre-incubation of the oligonucleotides with 1% dimethylsulphate (DMS), inducing methylation at the N7 position of guanine and preventing the Hoogsteen hydrogen bonding between N7 and the C2 amino group of neighbouring guanines (48) (Supplementary Figure S2A). Upon methylation, both Task3 WT (GGN)_13_ and NRAS WT G4 exhibited reduced molar ellipticity ([θ]) at the λ_max_ of 210 and 261 nm (Figure 1E and Supplementary Figure S2B respectively), indicating reduced G4 formation. However, the complementary base sequences and duplex controls showed stabilisation and increase in molar ellipticity at their respective λ_max_ that do not correspond to G4 formation (Supplementary Figure S2C–E). These data therefore suggest a dependency of Hoogsteen guanine–guanine hydrogen bonding on the structural conformations observed at 150 mM K^+^ for Task3 WT (GGN)_13_ and NRAS WT G4. Further, we observed that Task3 WT (GGN)_13_ G4 formation was independent of oligonucleotide concentration through no change in CD signature of between 1 and 50 μM oligonucleotides, indicative of an intramolecular G4 as opposed to a multi-strand association dependent on oligonucleotide concentration (data not shown). The G4 structure observed was also maintained when in the presence of the 11 nt upstream and 10 nt downstream of the Task3 5′ UTR (GGN)_13_ repeat (60 nt total length), and so formed within a wider sequence context (Supplementary Figure S3A).

Unlike duplex structures, G4 formation is dependent on the availability of monovalent cations to stabilise the central electronegative pore (49). To test the ionic dependency of Task3 (GGN)_13_, we compared G4 formation under different ionic conditions, either K^+^ to permit G4 formation or Li^+^ to inhibit G4 formation. At 150mM K^+^, Task3 (GGN)_13_ forms a parallel G4, exhibiting the CD λ_max_ of 210 and 261 nm (Figure 1F), whilst the duplex-forming control exhibits a λ_min_ of 210 and 250 nm and λ_max_ of 270–280 nm, typical of a B-form duplex (Supplementary Figure S3B). For Task3 WT (GGN)_13_ we observe a complete shift in spectral shape when folded in the presence of 150 mM Li^+^ (Figure 1F, dashed), exhibiting a trace more similar to that observed for the duplex control, indicating a clear absence of G4 formation in Li^+^ and a strong dependency on K^+^ availability for Task3 (GGN)_13_ G4 formation. As expected, the duplex sequence showed no ionic sensitivity, and its CD trace was maintained fully when shifting from K^+^ to Li^+^ (Supplementary Figure S3B). These results suggest that in the absence of K^+^ and presence of Li^+^, alternative molecular bonding is favoured for Task3 (GGN)_13_ instead of the guanine-guanine Hoogsteen bonding critical for G4 formation, a characteristic typical of G4 structures.

### Task3 G-quadruplex formation is K^+^ dependent

In order to further characterise the K^+^-dependency of G4 formation for Task3 (GGN)_13_, we analysed the CD spectra in the presence of different K^+^ concentrations from 5 to 150 mM K^+^. As before, we observe two distinct structural conformations at low vs high K^+^ concentrations, with spectra indicative of G4 formation only observed at 50 mM K^+^ and above. We observe an increase of approximately 10-fold in molar ellipticity at 210 and 261 nm between 50– and 150 mM K^+^ (Figure 1G). These data suggest a strong dependence on higher physiological K^+^ concentrations for Task3 G4 formation, and also the potential for Task3 (GGN)_13_ to form alternative structures, such as a duplex when folded discretely at low [K^+^] *in vitro*.

Conformational shifts between duplexes and G4s have been observed upon changes in ionic concentrations (50,51). Here however, we observe a conformational shift only when the ionic composition was discretely changed before folding of the oligonucleotides, with no change in Task3 spectra when oligonucleotides that had been pre-folded in 5 mM K^+^ were incubated with 150 mM K^+^ for 30 mins at RT (Supplementary Figure S3C). This suggests that the spectra observed for Task3 (GGN)_13_ at low [K^+^] or high [Li^+^] is not due to a single-stranded, unstructured oligonucleotide, instead due to a structured and stable non-G4 alternative structure as the presence of increased K^+^ is unable to overcome the structure formed at low [K^+^]. G4 formation can however be restored from these stable non-G4 alternative structures through addition of 150 mM K^+^ followed by 95°C denaturation and re-folding, further indicating the presence of a structured non-G4 alternative (Supplementary Figure S3C). G4 formation is also favoured for Task3 (GGN)_13_ when folded in the presence of equimolar concentrations of K^+^ and Li^+^ (Supplementary Figure S3D), suggesting that the lack of G4 formation is due to the absence of sufficient K^+^ ions as opposed to the presence of Li^+^ ions.

### Task3 G-quadruplex is thermostable under physiological conditions

G4s are highly thermostable under physiological ionic conditions (2), and so we characterised the thermostability of the Task3 (GGN)_13_ G4 in order to determine whether the G4 melting temperature was above physiological temperature. We performed a CD melt assay using 10 μM experimental oligonucleotides that were folded in 150 mM K^+^ and analysed within a Peltier cooled cell holder from 20 to 100°C, with spectra obtained at 5°C intervals.

Task3 (GGN)_13_ G4 exhibited a highly stable thermal profile, with no reduction in molar ellipticity at the λ_max_ until 75–80°C (Figure 1H). From these data it is evident that the G4 is thermostable under physiological K^+^, with an estimated melting temperature of between 75 and 80°C from the first derivative calculation of the melt curve for Task3 WT (GGN)_13_ (Data not shown). These data suggest that if G4 formation is maintained in the presence of 150 mM K^+^*in vivo*, the Task3 (GGN)_13_ G4 would remain folded at physiological temperature and could contribute to post-transcriptional regulation of the expression of Task3 channels.

### Task3 mRNA forms a stable G-quadruplex structure *in vivo*

**Figure 2.**
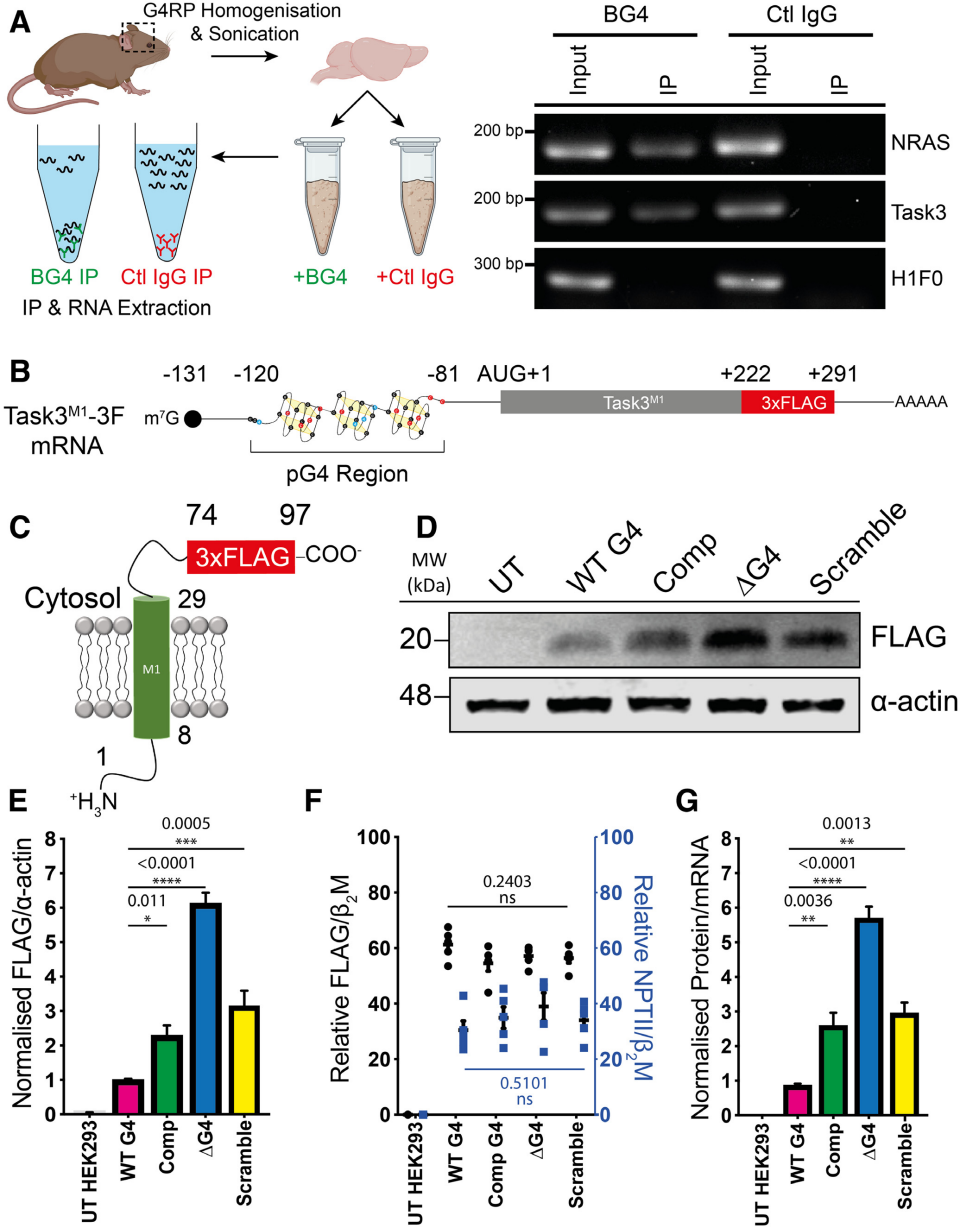
The Task3 5′ UTR G4 forms *in vivo* and inhibits translation of Task3-FLAG peptides *in vitro*. (**A**) Outline of experimental process for *in vivo* BG4 RNA immunoprecipitations from mouse brain homogenates using goat BG4 and control goat IgG antibodies (left) and endpoint RT-PCRs run on 1% agarose gel for input and IP fractions of BG4 and control goat IgG immunoprecipitations (right). (**B**) Schematic of the mRNA transcripts produced by the different Task3 mutant 5′ UTR constructs, possessing either the wild-type full length 5′ UTR or various mutant sequences including a complementary (CCN)_13_ substitution, a 39 nt deletion (ΔG4) or a 39 nt scramble substitution. (**C**) All constructs translate the identical 97 amino acid peptides (Task3^M1^–3F) allowing for comparison of different translation efficiencies that arise from different structural species present within Task3 5′ UTR. (**D**) Western blotting of HEK-293 lysates transfected with each Task3 mutant 5′ UTR construct. (**E**) Quantification of Task3^M1^-FLAG expression normalised to α-actin expression. (**F**) Mutant 5′ UTR Task3^M1^-FLAG mRNA transcript abundance normalised to endogenous HEK-293 β_2_M (Black) or the internal vector control NPTII normalised to HEK-293 β_2_M (Blue) for transfection efficiency control. (**G**) Relative translation efficiency attributed to each of the wild-type or mutant Task3 5′ UTR mRNA transcripts where Task3 WT G4 5′ UTR = 1. N = 5 biological replicates where error bars represent SEM. Statistical analyses were carried out by one-way ANOVA (E – *F*(3, 20) = 69.9, *P* < 0.0001, F – FLAG: *F*(3, 20) = 1.56, *P* = 0.2403, NPTII: F(3, 20) = 0.81, *P* = 0.5101. G – (*F*(3,20) = 50.01, *P* < 0.0001) with post-hoc Sidak's multiple comparison tests, *P*-values shown. UT = untransfected.

Since we observed G4 formation for the Task3 (GGN)_13_ repeat under physiological [K^+^] *in vitro* and at temperatures substantially greater than physiological temperatures, we next investigated whether G4 formation was maintained *in vivo* within Task3 mRNA. To investigate this, an RNA immunoprecipitation was employed using the G4-binding BG4 antibody (52,53). Cerebral cortical tissue was isolated from adult mice, homogenised and sonicated in G4RP buffer before being split into two equal fractions for control IgG and BG4 RNA IPs (Figure 2A, left). RNAs were isolated from the input and IP fractions and analysed through RT-PCR for the presence of Task3 mRNA alongside NRAS mRNA as a G4 containing positive control and H1F0 mRNA as a non-G4 containing negative control (Figure 2A, right). All target mRNAs of interest were detected in the input fractions for both the BG4 and control IgG fractions. As expected, RNA concentrations in the control IgG IPs were negligible and no mRNAs of interest were detected in this fraction. Both NRAS and Task3 mRNAs were detected within the BG4 IP fraction, whilst H1F0 mRNA was not detected, suggesting a specific pull-down of G4 containing mRNAs within this fraction. The same results were replicated in human neuroblastoma (SH-SY5Y) cells *in vitro* (not shown). These data suggest that Task3 mRNA is forming a G4 structure in human cells and in the adult mouse brain *in vivo*.

### Task3 5′ UTR G-quadruplex inhibits translation of Task3-FLAG reporter constructs

To study the influence of the 5′ UTR (GGN)_13_ G4 on the translation efficiency of Task3, we generated reporter constructs possessing WT or mutated regions within the 5′ UTR G4 region (Figure 2B) that all produce identical 3x FLAG-tagged peptides termed Task3^M1^-3F (Figure 2C). The product of translation consisted only of the N-terminal cytoplasmic and first transmembrane domains in order to avoid formation of functional Task3 K^+^ channels that would alter intracellular K^+^ concentration. Upstream of the coding sequence, the four experimental constructs used differ only in the G4-forming (GGN)_13_ region between positions –120 and -81 of the AUG initiation site. The WT construct contains the full 131 nt 5′ UTR, the complementary (Comp) control contains a (CCN)_13_ repeat substitution within the 39 nt G4-forming region, the ΔG4 mutant contains a complete deletion of the 39 nt G4-forming region whilst maintaining the rest of the 95 nt 5′ UTR, and the scramble mutant containing a 39 nt GC rich scramble sequence as a secondary structure-free length-matched control sequence (Figure 1B and Supplementary Figure S1A).

HEK293 cells were transfected with the FLAG-tagged reporter constructs and protein lysates were analysed through Western blotting 48 hours post-transfection (Figure 2D). Task3^M1^-3F expression was normalised to α-actin and quantified (Figure 2E), and a significant degree of expressional control was elicited in the presence of the 5′ UTR G4. We observed a significant increase in expression of Task3^M1^-3F by ∼6-fold upon deletion of the G4-forming (GGN)_13_ repeat (ΔG4) (WT G4, 1.065 ± 0.041; ΔG4, 6.125 ± 0.313, *P* = 0.0001), thus suggesting it acts as a key regulatory mechanism of controlling rates of Task3 translation. Replacing the G4-forming repeat with the GC-rich length-matched scramble sequence led to a ∼3-fold increase in Task3^M1^-3F expression in comparison to the WT G4-forming (GGN)_13_ repeat (WT G4, 1.065 ± 0.041; Scramble, 3.127 ± 0.465, *P* = 0.0005). This scramble sequence formed no distinct structure *in vitro* (Supplementary Figure S1A) and so these data suggest that the overall unstructured length of the 5′ UTR indeed has an effect on translational efficiency. Thus, the true degree of translational repression elicited by formation of a G4 within the (GGN)_13_ region is overestimated when comparing only to the ΔG4 5′ UTR mutant. The complementary (CCN)_13_ repeat elicits only a partial ∼2.5-fold relief in translation repression compared to the WT G4 construct (WT G4, 1.065 ± 0.041; Comp, 2.285 ± 0.295, *P* = 0.011) which may be the result of an alternate secondary structure such as suggested in Figure 1C. More likely, there is a length-dependent effect as the differences observed between the complementary and scramble sequences is not statistically significant (Scramble, 3.127 ± 0.465; Comp, 2.285 ± 0.295, *P* = 0.3179).

To confirm that the observed increase in Task3^M1^-3F expression was due to G4-induced translational repression as opposed to transcriptional repression, we quantified levels of Task3^M1^-3F mRNA via qPCR (Figure 2F). No significant variations were observed for Task3^M1^-3F mRNA when normalised to the endogenous HEK293 beta-2-microglobulin (β2M) (*F*(3, 20) = 1.56, *P* = 0.2403), indicating no significant changes in levels of transcription. Similarly, no significant variation in levels of neomycin phosphotransferase 2 (NPTII) mRNA, an internal vector control gene expressed from the transfected plasmids, were detected when normalised to endogenous β2M mRNA (*F*(3, 20) = 0.81, *P* = 0.5101), suggesting consistent levels of transfection for each construct (Figure 2F). These data suggest that all constructs possess no significant difference in transfection rate nor levels of Task3^M1^-3F transcription. The data from Figure 2E and F allowed for comparison of relative mRNA to protein levels for Task3^M1^-3F to inform us of the translation efficiency for each construct (Figure 2G). These data further suggest that the increase in Task3^M1^-3F expression upon deletion and mutation of the G4-forming (GGN)_13_ repeat is due the loss of the translationally repressive moiety.

### Overexpression of the G-quadruplex-specific helicase DHX36 partially relieves the G-quadruplex translation repression of Task3

**Figure 3.**
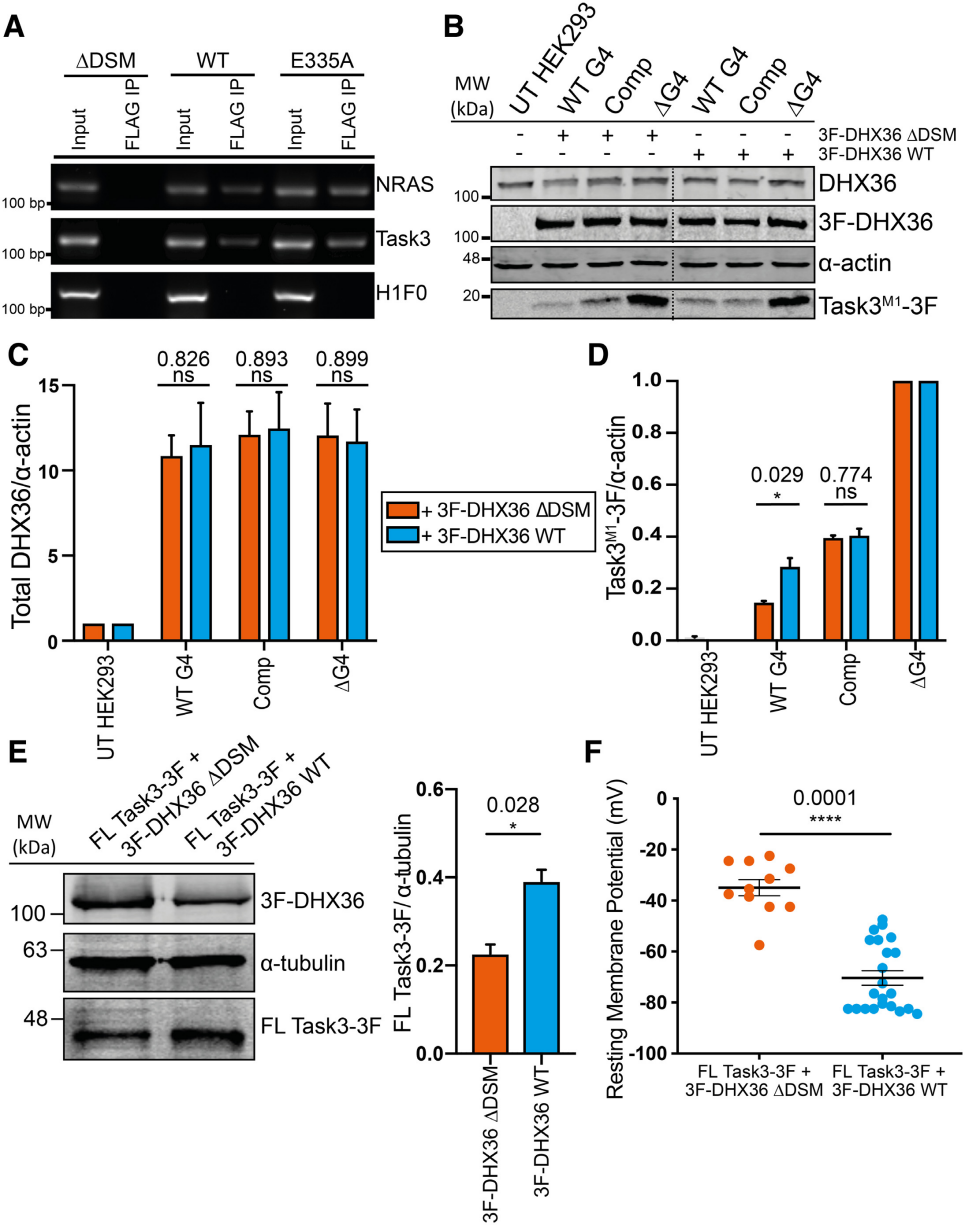
The G4 helicase DHX36 binds to Task3 mRNA and partially relieves the G4-mediated translation repression. (**A**) Endpoint RT-PCR of 3F-DHX36 mRNA targets isolated through FLAG immunoprecipitation in transfected HEK293 cells. (**B**) Western blotting of HEK-293 lysates co-transfected with each Task3 mutant 5′ UTR construct and either the G4 resolving helicase DHX36 or a non-G4 resolving mutant (DHX36 ΔDSM). (**C**) Quantification of endogenous and exogenous DHX36 expression for each co-transfection condition normalised to α-actin expression. (**D**) Quantification of Task3^M1^–3F expression normalised to α-actin expression. In each co-transfection condition, Task3^M1^-3F expression values for the Task3 5′ UTR mutant constructs were normalised to the ΔG4 control. (**E**) Western blotting of HEK293 lysates transfected with FL Task3–3F as well as either 3F-DHX36 WT or ΔDSM (Left) with exogenous FL Task3–3F expression quantification normalised to α-tubulin (Right). (**F**) Whole cell patch clamp recordings of HEK293 cells co-transfected with FL Task3–3F as well as either 3F-DHX36 WT or ΔDSM. Each data point represents an individual neuron. *N* = 4 biological replicates where error bars represent SEM. Statistical analyses were carried out by unpaired two-tailed *t*-tests, *P*-values shown. UT = untransfected.

To date, there have been many G4-resolving helicases identified. Of these, the DEAH-box helicase 36 (DHX36) is the best-characterised G4-RNA resolving helicase with its structure, function, and molecular kinetics well-studied (54–57). Data for Task3 are lacking in previous genome- and transcriptome-wide DHX36 studies, and so we sought to investigate a potential interaction between the G4 helicase DHX36 and the G4-containing Task3 mRNA through immunoprecipitation of N-terminally FLAG-tagged full-length DHX36 constructs (3F-DHX36). HEK293 cells were transfected with the WT 3F-DHX36 alongside a mutant DHX36 lacking the DHX36-specific domain (DSM) responsible for G4-binding (3F-DHX36 ΔDSM) or a point-mutant that catalytically inactivates the helicase and increases the binding time to G4 substrates (3F-DHX36 E335A) (58). FLAG IPs were carried out in G4RP buffer as previously described. RNAs from the input and FLAG IP fractions were isolated and investigated through RT-PCR for the presence of Task3 mRNA alongside NRAS mRNA as a G4 containing positive control and H1F0 mRNA as a non-G4 containing negative control (Figure 3A). As expected, FLAG IP of 3F-DHX36 ΔDSM led to no detection of NRAS, Task3 or H1F0 mRNA. However, NRAS and Task3 mRNA were both detected through FLAG IP of 3F-DHX36 WT and E335A, whilst H1F0 was not detected in either of these fractions. These data suggest that endogenous Task3 mRNA was indeed a substrate for 3F-DHX36 in HEK293 cells, which coupled with BG4 IP data showing that Task3 mRNA is forming a G4 structure *in vivo* suggests DHX36 may have some involvement in the G4-dependent regulation of Task3 expression.

To assess whether overexpression of DHX36 in could relieve the G4 mediated translation repression we observe for Task3^M1^ mRNA, HEK293 cells were subsequently co-transfected with Task3^M1^-3F WT or mutant 5′ UTR constructs and either 3F-DHX36 WT or the non-G4 binding ΔDSM mutant to investigate the influence of DHX36 on expression of Task3. HEK293 cells transfected with 3F-DHX36 constructs drove an average increase in DHX36 expression of between 10–12-fold (Figure 3B and C), with no significant difference in the expression levels of WT or ΔDSM 3F-DHX36 constructs. Cell viability of HEK293 cells was not affected by transfection of these constructs at either 24 or 48 h timepoints (Supplementary Figure S4A and B). Expression of Task3 for each condition was normalised to the Task3^M1^-3F ΔG4 to compare translation efficiencies of G4 vs non-G4 mRNA in the presence of 3F-DHX36 WT or ΔDSM (Figure 3D). When co-expressed with 3F-DHX36 ΔDSM, Task3^M1^-3F maintained a similar expression pattern for the 5′ UTR variants as was previously observed in Figure 2D and E. However, upon co-transfection with 3F-DHX36 WT, we observed a 2-fold increase in the normalised expression of Task3^M1^-3F possessing the wild-type full-length 5′ UTR leader (0.145 ± 0.010; 0.283 ± 0.034, *P* = 0.029). In contrast, when the 5′ UTR (GGN)_13_ sequence was mutated to (CCN)_13_, and co-transfected with 3F-DHX36 WT, we observed no significant change in Task3^M1^-3F expression in comparison to the co-transfection with the 3F-DHX36 ΔDSM mutant (0.394 ± 0.021; 0.403 ± 0.027, *P* = 0.774). No significant differences in Task3^M1^-3F or 3F-DHX36 mRNA were detected between transfection conditions (Supplementary Figure S4C). These data demonstrate that 3F-DHX36 selectively increases the translational efficiency of the Task3^M1^ mRNA containing the WT G4 sequence.

To investigate whether this effect was maintained endogenously and within the context of the full-length Task3 mRNA sequence, we analysed the expression of endogenous Task3 upon overexpression of 3F-DHX36 WT in comparison to 3F-DHX36 ΔDSM (Supplementary Figure S4D). We observe that upon transfection of 3F-DHX36 WT, endogenous Task3 expression was increased by an average of ∼1.25-fold when compared to the 3F-DHX36 ΔDSM control (0.407 ± 0.043; 0.543 ± 0.025, *P* = 0.035). These data suggest that as shown in Figure 3A, endogenous Task3 mRNA is a substrate for DHX36 through G4 binding and that this increases translational efficiency of endogenous Task3 mRNA.

### Increasing Task3 expression through DHX36 G-quadruplex unwinding Increasing Task3 expression through DHX36 G-quadruplex unwinding hyperpolarises HEK293 cells

Since overexpression of DHX36 leads to an increase in endogenous Task3 expression in HEK293 cells (Supplementary Figure S4D), we sought to investigate if this overexpression would lead to a Task3 dependent alteration in the membrane potential of HEK293 cells from increased K^+^ leak currents. We predicted that an increase in plasma membrane expression of Task3 K^+^ channels would allow us to detect a negative shift in membrane potential (hyperpolarisation), and so we measured membrane potentials of HEK293 cells transfected with either 3F-DHX36 WT or ΔDSM using whole-cell patch clamp recordings. When compared to cells transfected with 3F-DHX36 ΔDSM, we observe a significant hyperpolarisation for cells transfected with 3F-DHX36 WT (−23.83 ± 1.94 mV; −41.25 ± 2.45 mV, *P* = 0.0012) (Supplementary Figure S4E). However, from these data we cannot attribute this effect on resting membrane potential to an increase in Task3 expression alone as DHX36 may have additional mRNA substrates that upon translation also influence resting membrane potential.

To investigate this further, we co-transfected a full-length Task3 construct possessing the WT 5′ UTR (FL Task3-3F) with either 3F-DHX36 WT or ΔDSM, with the intent of increasing Task3 mRNA levels to drive a larger hyperpolarisation and identify whether the shift observed with 3F-DHX36 WT was at least in part driven by the action of increasing Task3 expression. Whilst overexpression of 3F-DHX36 WT modestly increased endogenous Task3 expression by ∼1.25-fold in comparison to the ΔDSM control (Supplementary Figure S4E), an overexpression of both DHX36 and Task3 mRNA further increased FL Task3-3F expression by ∼2-fold when compared to the ΔDSM control (0.226 ± 0.021; 0.391 ± 0.026, *P* = 0.028) (Figure 3E), similar to the level of increase observed for the protein expression of Task^M1^-3F (Figure 3B and D). This was accompanied by a significantly larger hyperpolarisation for FL Task3-3F + 3F-DHX36 WT compared to FL Task3-3F + 3F-DHX36 ΔDSM (−34.95 ± 10.41 mV; −70.36 ± 13.27 mV, *P* = 0.0001) (Figure 3F) suggesting that the hyperpolarisation of HEK293 cells upon DHX36 overexpression are at least in part driven by increased incorporation of Task3 K^+^ leak channels into the plasma membrane.

### Distal localisation of Task3 mRNA is dependent on presence of the 5′ UTR G-quadruplex

**Figure 4.**
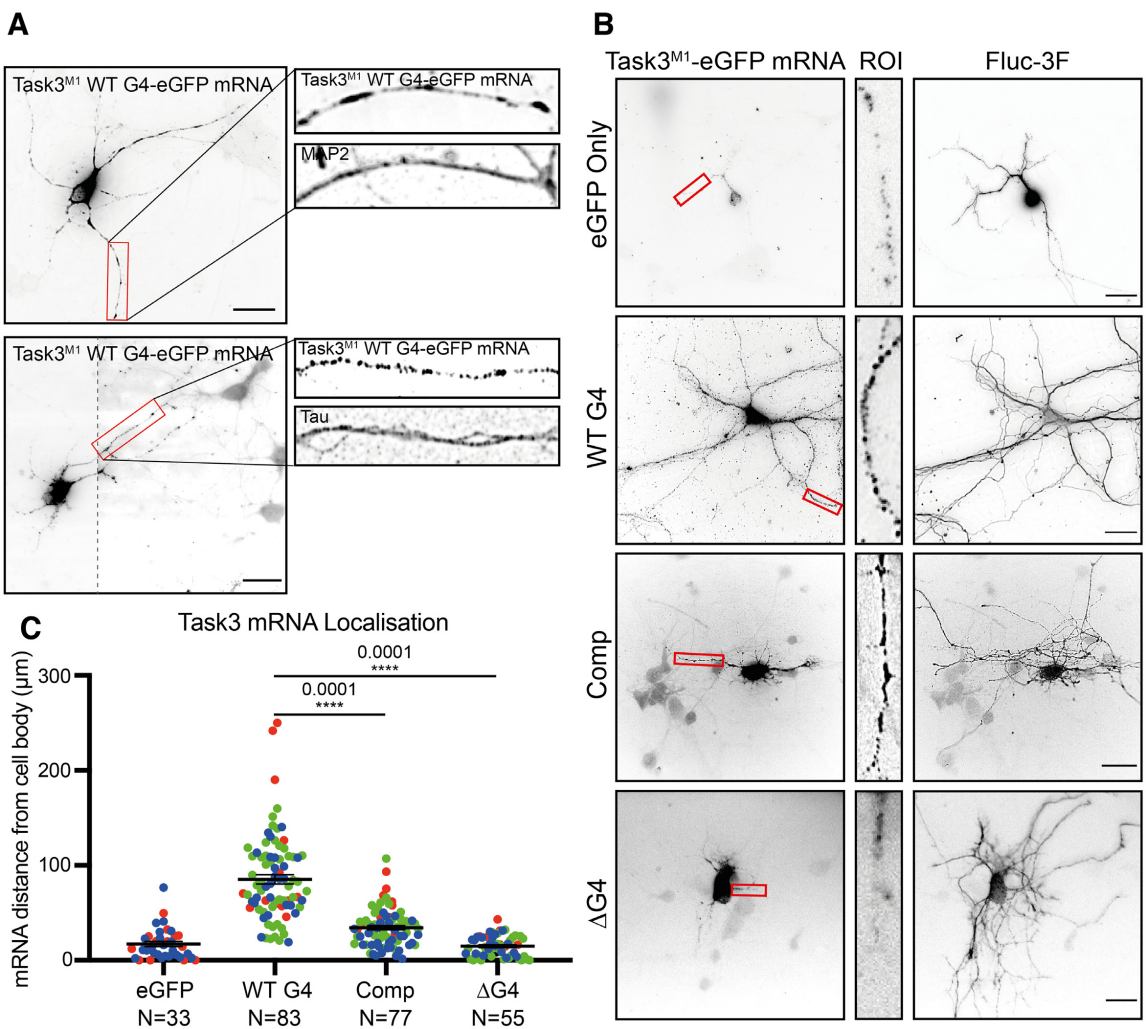
Task3 5′ UTR (GGN)_13_ repeat is required for distal neurite localisation of Task3 mRNA. (**A**) RNA FISH of DIV10 embryonic murine cortical neurons transfected with WT G4 Task3^M1^-eGFP. Cells were hybridised with anti-eGFP probes and anti-MAP2/Tau primary antibodies to determine neurite localisation of exogenous Task3 mRNA. Image 2 was taken at two exposures to define mRNA granules in both the cell body and neurites with the respective images merged (dotted line). Original un-edited images available in Supplementary Figure S5. (**B**) RNA FISH of transfected DIV10 embryonic murine cortical neurons co-transfected with mutant G4 5′ UTR Task3^M1^-eGFP and Fluc-3F as a soluble whole-cell marker. Cells were hybridised with anti-eGFP probes and anti-FLAG M2 primary antibody showing localisation of exogenous mutant G4 5′ UTR Task3^M1^-eGFP mRNA in comparison to whole-cell staining. (**C**) The distance of exogenous mutant G4 5′ UTR Task3^M1^-eGFP mRNA from the cell body, quantified in Fiji for cells possessing neurites ≥100 μm in length. Number of neurites analysed is shown from three blinded biological replicates (coloured groups), where error bars represent SEM. Statistical analyses were carried out by one-way ANOVA (*F*(3, 247) = 89.01, *P* < 0.0001), with post-hoc Sidak's multiple comparison tests, *P* values shown. Scale bar = 20 μm.

There is increasing evidence that G4s are involved in regulating local translation of neuronal mRNAs through permitting the localisation of mRNA to these distal compartments (19,21,59). We therefore wanted to investigate whether in addition to regulating translation, the 5′ UTR G4 also mediated subcellular localisation of Task3 mRNA. To investigate this, the mutant 5′ UTR Task3^M1^ constructs were used as in previous experiments, with the C-terminal FLAG tag substituted for eGFP to provide a longer target sequence for RNA FISH probes, as well as to distinguish between endogenous Task3 mRNA and the experimental G4 exogenous Task3^M1^ transcripts. Subcellular localisation of exogenous WT G4 Task3^M1^-eGFP mRNA was initially determined within murine embryonic cortical neurons through simultaneous FISH and immunocytochemistry for the axonal and dendritic markers, tau and MAP2 respectively (Figure 4A). Exogenous WT G4 Task3^M1^-eGFP mRNA was localised to both axons and dendrites, where mRNA co-localized with both tau and MAP2. We subsequently sought to identify how mutating the 5′ UTR (GGN)_13_ G4 repeat influenced neuronal subcellular localisation of Task3 mRNA to determine whether the Task3 5′ UTR G4 was responsible for neurite localisation.

To address this, the mutant constructs were co-transfected into murine embryonic cortical neurons with a FLAG-tagged firefly luciferase reporter (Fluc-3F) to enable the staining of whole individual transfected cells within a dense neuronal network. Cells were fixed and co-hybridised with anti-eGFP mRNA probes and anti-FLAG M2 primary antibody (Figure 4B). Transfected cells possessing neurites ≥100 μm in length were then subsequently analysed for the distance that exogenous Task3^M1^-eGFP mRNA could be detected from the cell body (Figure 4C).

The Fluc-3F staining indicates that extensive neurite outgrowth was achieved for embryonic murine cortical cells in culture. However, despite consistent levels of neurite outgrowth in each condition, the distance of Task3^M1^-eGFP mRNA reactivity from the cell body of each neuron varied considerably between each of the 5′ UTR G4 region mutants. One-way ANOVA analysis indicates a significant effect of the G4 region manipulation on mRNA distance from the cell body, *F*(3, 247) = 89.01, *P* < 0.0001. Further post-hoc pairwise comparisons showed that the ΔG4 Task3^M1^-eGFP mRNA showed no significant difference to the eGFP only control, yielding averages of 14.84 ± 1.46 μm and 17.15 ± 2.75 μm respectively (*P* = 0.99). In contrast, Comp Task3^M1^-eGFP mRNA showed slightly elevated distances for mRNA detection from the cell body in comparison to the eGFP control, with an average of 34.34 ± 2.26 μm (*P* = 0.02). However, WT G4 Task3^M1^-eGFP mRNA showed the greatest distance of detection from the cell body out of all conditions, with an average of 85.23 ± 4.83 μm (*P* < 0.0001). These data suggest that the presence of either the (GGN)_13_ or (CCN)_13_ repeat within the 5′ UTR was sufficient to drive a significant change in neurite mRNA localisation, with the presence of the (GGN)_13_ G4 forming sequence required for full distal neurite localisation. When comparing within the G4 region mutants, both complementing and deleting the (GGN)_13_ repeat yielded highly significant reductions in distal neurite mRNA localisation when compared to the WT G4, further suggesting that the Task3 WT (GGN)_13_ G4 formation is required for distal Task3 mRNA localisation. Interestingly however, neurite localisation is not completely lost when complementing the (GGN)_13_ repeat as it is when deleting the repeat. As inferred from Figures 1 and 2, it is likely that the (CCN)_13_ repeat forms an alternative secondary structure such as a duplex/stem-loop or intercalated motif (i-motif) due to the CD spectra and mild translational repression observed. This could also be influencing subcellular localisation of the transcripts through a distinct mechanism to that of G4 driven localisation, potentially mediated by an alternative family of RNA binding proteins (RBP).

### Task3 protein and mRNA are distally localised to neurites in murine embryonic cortical neurons

**Figure 5.**
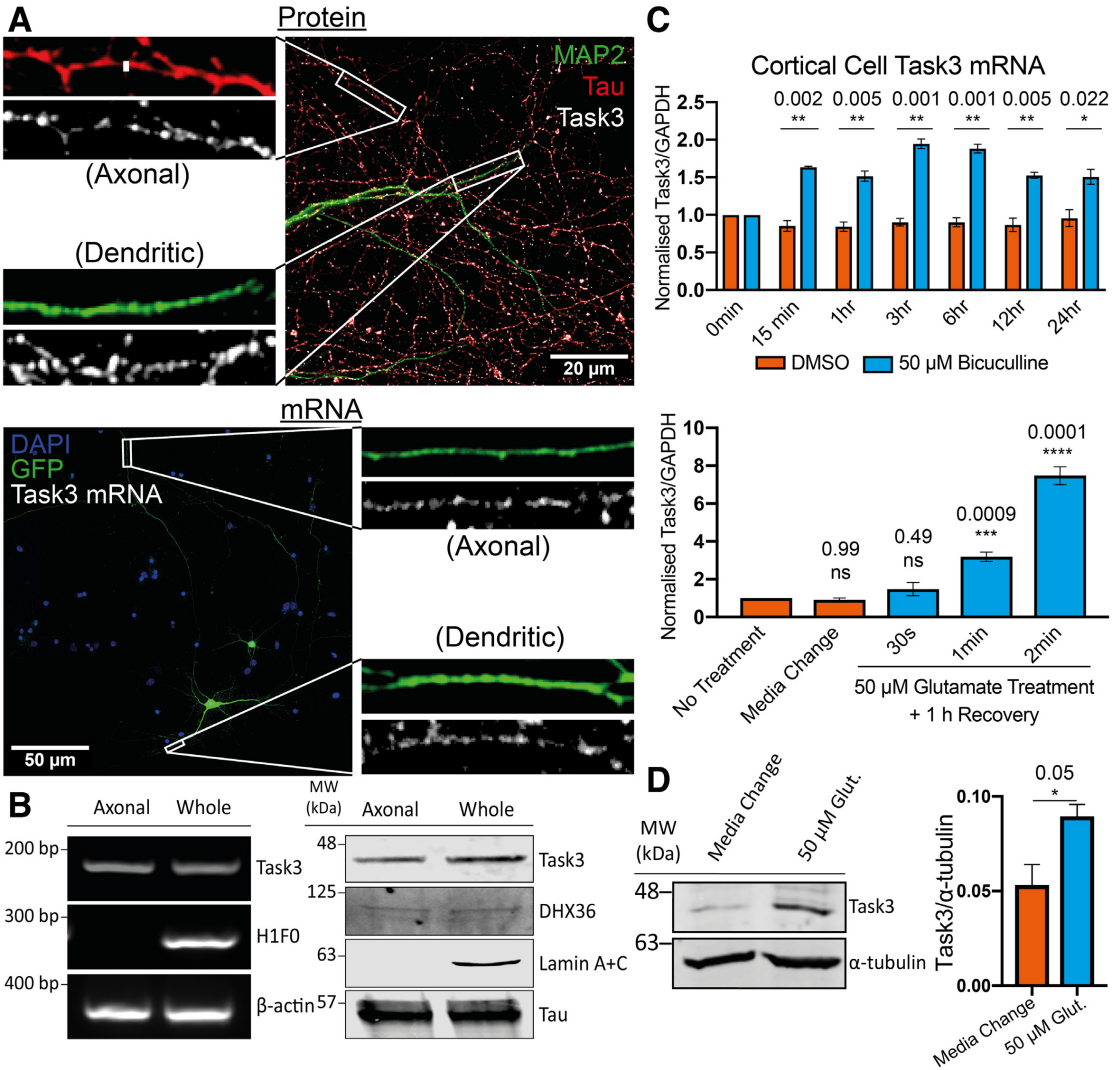
Endogenous Task3 protein and mRNA are localised to distal neurites and upregulate in response to neuronal activity. (**A**, top) Immunocytochemistry of wild-type DIV10 embryonic murine cortical neurons stained for the endogenous dendritic and axonal markers, MAP2 (Green) and Tau (Red) respectively, as well as endogenous Task3 protein (white). (A, bottom) RNA Fluorescence *in situ* Hybridisation (FISH) of eGFP transfected DIV10 embryonic murine cortical neurons hybridised with anti-Task3 mouse probes showing endogenous Task3 mRNA from a singular GFP transfected neuron in distal neurites. (**B**) Western blot (left) and endpoint RT-PCR (right) analyses for specific protein and mRNA species’ from DIV10 primary embryonic cortical neurons cultured in Corning^®^ Transwell^®^ inserts for the isolation of whole neuron vs axonal proteins and mRNAs. (**C**) Total Task3 mRNA levels from DIV10 embryonic cortical neurons treated with 50 μM bicuculline or DMSO over a 24 h period (top), as well as with 50 μM glutamate for 30s, 1min, 2min or a no-treatment control media change with a 1h recovery period (bottom). (**D**) Western blotting of whole cortical cell lysates after 2 min treatment with 50 μM glutamate or no-treatment control media change with a 1 h recovery period (Left) with quantification of endogenous Task3 expression normalised to α-tubulin (Right). *N* = 4 biological replicates where error bars represent SEM. Statistical analyses were carried out by one-way ANOVA (C (bottom): (*F*(4, 10) = 90.60, *P* < 0.0001) with post-hoc Sidak's multiple comparison tests or by unpaired two-tailed *t*-tests; C (top) and D, *P*-values shown.

With the identification of a parallel G4 structure in the 5′ UTR of Task3 mRNA that regulates translational efficiency and localisation of exogenous Task3^M1^ mRNA, we finally wanted to identify how endogenous Task3 protein and mRNA is localised within the context of the full-length endogenous transcripts in neurons. Task3 protein localisation was investigated through immunocytochemistry of DIV10 primary cortical neurons using antibodies against Task3 as well as the axonal and dendritic markers Tau and MAP2 respectively (Figure 5A, top). Task3 protein localisation overlaps with both Tau and MAP2 immunoreactivity suggesting localisation distally within both axonal and dendritic projections of neuronal cells, consistent with previous observations in rat motor neurons (60).

To investigate subcellular localisation of endoenous Task3 protein mRNA in cortical neurons, we used RNA FISH probes targeting mouse Task3 mRNA. Wild type murine embryonic cortical cells were cultured *in vitro* and transfected with eGFP to highlight individual cells within dense neuronal cultures. When tracing neurites from a single eGFP transfected neuron, endogenous Task3 mRNA was observed to be present in both the distal axonal and dendritic projections (Figure 5A, bottom).

To confirm the presence of Task3 protein and mRNA in distal axonal projections, cortical cells were also cultured in Transwell^®^ inserts (Figure 5B). This allows separation of axonal projections by allowing the growth of a mixed neurite population on the apical membrane face, whilst only an axonal population on the basal membrane face due to constraints in width of the membrane pores (39). RNA and protein extracted from both sides of the Transwell^®^ inserts were analysed through RT-PCR (Figure 5B, left) and western blotting (Figure 5B, right) for the presence of Task3 and DHX36. The detection of nuclear-localised lamin protein and histone 1 (H1F0) mRNA in only the whole-cell mixed preparation, as well as the detection of Tau protein and β-actin mRNA in both axonal and whole-cell preparations confirms clean isolation of axon-only samples with no cross-membrane contamination. From this, Task3 mRNA and protein were also detected in both preparations respectively, confirming the presence of Task3 mRNA and protein in the distal axon. The presence of Task3 mRNA in both axonal and dendritic projections correlates with that observed for exogenous Task3^M1^ mRNA, suggesting that the G4-mediated control of exogenous mRNA localisation may too be seen for the endogenous Task3 mRNA. Similarly, the presence of endogenous Task3 mRNA distally from the cell body suggests a potential contribution of local translation to Task3 expression.

### Both Task3 mRNA and protein are upregulated in response to neuronal activity

Neuronal activity plays a key role in regulating the spatiotemporal control of local translation that underpins dynamic plasticity at synapses (61–63). Since Task3 mRNA is localised to distal neurites, we sought to examine the role that neuronal activity may play in Task3 mRNA levels, localisation and protein expression. Neuronal cultures contain a finely balanced network of excitatory and inhibitory neurons, with inhibitory neurons becoming responsive at DIV7 and full maturation of network connections at DIV12-13 (64). As a result of this heterogeneous cell population, different pharmacological approaches can be utilised to elicit neuronal activity, with differing intensities and functional outcomes. Bicuculline is a GABA_A_ receptor antagonist that increases neuronal spiking and synaptic protein expression (65), whilst glutamate activates both metabotropic and ionotropic glutamatergic receptors including AMPA and NMDA ion channels (66).

We treated wild-type DIV13 murine embryonic cortical neurons with 50 μM bicuculline or a DMSO vehicle control over a 24 h period to induce a low-level increase in basal neuronal spiking through a blockade of endogenous inhibitory circuits (Figure 5C, top). Similarly, we also treated DIV13 murine embryonic cortical neurons with 50 μM glutamate for 30 s, 1 and 2 min with a 1 h recovery period, to induce a short period of intense neuronal activation. RNA was extracted and analysed through RT-qPCR for levels of Task3 mRNA relative to GAPDH (Figure 5C, bottom). We find that both bicuculline and glutamate are able to induce global transcription of Task3 mRNA to differing degrees. Treatment with a DMSO vehicle control induced no change in Task3 mRNA levels, whereas treatment with 50 μM bicuculline induced a modest but consistent increase in Task3 mRNA of between 1.5- and 2-fold over a 24 h period of stimulation. However, treatment with 50 μM glutamate for just 60 s followed by a 1 h recovery period was sufficient to elicit a 3-fold increase in Task3 mRNA, and treatment for 120 s elevated Task3 mRNA levels between 7- and 8-fold.

In addition to upregulation of mRNA transcription within the nucleus, neuronal activity is also a key driver of regulated local protein synthesis in distal neuronal compartments (67). Given that we observe an increase in levels of Task3 mRNA during activity, investigated whether this corresponded with an increase in Task3 protein expression that could regulate K^+^ leak currents during periods of prolonged neuronal activity. To analyse this, we treated primary cortical neurons with 50 μM glutamate for 2 min followed by 1 h recovery, as in the previous experiments for mRNA quantification. Cortical cell lysates were analysed through Western blotting for Task3 expression with 50 μM glutamate compared to a media change control (Figure 5D), and we observe an increase in Task3 protein translation of ∼1.6-fold upon treatment with 50 μM glutamate. These data indicate that both Task3 mRNA and protein are both responsive to neuronal activity, with Task3 upregulation likely to play a role in maintaining membrane conductance to K^+^ during the neuronal activity changes that may associated with synaptic plasticity or neuroprotection during excitotoxicity.

## DISCUSSION

*In silico* and bioinformatic analyses predict the presence of over 10 000 potential G4 (pG4) sequences within the human transcriptome (14) and there is now a wealth of evidence in support for G4 formation within both DNA and RNA *in vivo*. Visualisation of their presence within both the nuclei and cytoplasm of cells has come through the generation of the G4 specific antibody, BG4 (52) as well as live cell single-molecule imaging of G4s using G4-specific fluorescent ligands showing their fluid molecular dynamics within a cellular context (68,69). Similarly, the effect that these G4-specific ligands have on genomic stability as well as transcriptional and translational activity at pG4 regions also suggests the maintenance of these structures *in vivo* (9).

In this study, we sought to investigate the structure of and functional influence of a 5′ UTR (GGN)_13_ repeat on the localisation and regulated expression of Task3 mRNA. Through biophysical, biochemical, electrophysiological and cell culture approaches, we have gathered substantial evidence to suggest that this (GGN)_13_ repeat forms a stable parallel G4 structure both *in vitro* and *in vivo* that inhibits translation of the two-pore domain potassium leak channel Task3 mRNA. Translational repression can be relieved endogenously and exogenously by the well-characterised G4 helicase, DHX36. A negative shift of the membrane potential indicates that protein expression and plasma membrane expression also take place and result in modification of K^+^ leak current. Further, our data suggest that the presence of this 5′ UTR G4 is necessary for correct neurite localisation of Task3 mRNA in primary neuronal cultures, providing a new insight into potential mechanisms controlling the regulated expression of K2P channels as well as the roles of 5′ UTR G4 structures.

G4s are stabilised by monovalent cations within the central electronegative pore, due to inward orientation of the electron-rich carbonyl groups from each guanine. K^+^ shows the strongest co-ordinating ability due to its ionic radius, with Na^+^ showing weak stabilisation and Li^+^ unable to stabilise G4s (49,70). K^+^ is the most abundant exchangeable cation in the body, with intracellular concentrations of K^+^ at ∼150 mM through the action of different K^+^ transporters/channels (71), so we therefore based our biophysical experiments around optimal intracellular K^+^ concentrations of 150 mM. Through CD, Task3 (GGN)_13_ exhibited all of the expected classical G4 characteristics, including a strong dependency on K^+^ concentration, an inhibition of G4 formation through DMS methylation at the N7 position of guanine, and strong thermostability *in vitro*. These characteristics were lost in the absence of K^+^ and also when both a complemented (CCN)_13_ repeat and GC-rich length-matched sequences were investigated.

The CD spectra for the WT (GGN)_13_ sequence in the absence of K^+^, as well as the complemented (CCN)_13_ sequence at physiological [K^+^] both showed similarities to that observed for a duplex-forming control. These data suggest that under physiological [K^+^] conditions, Task3 (GGN)_13_ forms a stable parallel G4, however under non-physiological K^+^ conditions, it adopts an alternate structure, likely a duplex or stem-loop structure in the absence of the stabilising K^+^ ions. This was not observed for the NRAS WT G4, where G4 formation was maintained under all conditions except upon DMS-induced N7 methylation of guanine, suggesting that this is likely due to the topological arrangement of bases and the repeating nature of (GGN)_13_, not just due to G4 formation. *In vivo* this may suggest a more complex equilibrium between the two structures depending on cellular ionic conditions, a process that has previously been observed for other GC rich G4 structures (50).

RNA immunoprecipitation from adult mouse brain homogenates using the G4-RNA-binding antibody BG4 suggested that Task3 mRNA indeed contains a G4 structure *in vivo* (Figure 2A). As the (GGN)_13_ sequence readily shows stable G4 formation *in vitro* (Figure 1) and there are no sequences within Task3 mRNA that show greater potential for G4 formation, we attributed this to G4 formation within the (GGN)_13_ repeat sequence.

When investigating the G4 influence on Task3 expression, several considerations were made to ensure a degree of physiological representation. Codon selection and amino acid composition is known to influence translation rate and efficiency post-initiation (72). Similarly, overexpression of K^+^ leak channels perturbs cellular signalling and K^+^ conductance (73), which could therefore influence G4 folding and give a non-physiological representation of the action of the Task3 5′ UTR G4. To overcome this, we designed Task3 constructs that translate identical peptides possessing only the N-terminal cytoplasmic and first transmembrane domains with a C-terminal triple FLAG tag. This avoided formation of functional Task3 K^+^ channels that would alter intracellular K^+^ concentrations, whilst maintaining physiological codon selection post-translation initiation. We find that the G4 located in the 5′ UTR of Task3 elicited ∼6-fold decrease in translation of Task3^M1^–3F peptides compared to when the (GGN)_13_ repeat was deleted, and a 3-fold decrease when compared to a GC-rich length-matched scramble sequence in its place, a similar effect to that originally observed for the NRAS proto-oncogene 5′ UTR G4 (12). G4s are prevalent within oncogenes, which has been suggested as a mechanism to buffer the overexpression of oncogenic proteins (74). The overexpression of Task3 and other K^+^ leak channels alters cellular K^+^ conductance and has been implicated in a number of cancers (75,76), suggesting this 5′ UTR G4 may control the regulated low level expression of Task3 required for normal physiological expression. In contrast, dysfunction and knockdown of Task3 leak channels leads to impaired neuronal migration of cerebral cortical neurons in the developing murine brain and the maternally imprinted intellectual disability Birk-Barel mental retardation (34,77). Taken together, these data suggest that Task3 expression sits within a narrow physiological window, which the 5′ UTR G4 may be central to regulating.

DHX36 (also known as RHAU and G4R1) is a well-characterised G4-specific helicase and has been shown to bind preferentially to G4 and G-rich sequences on over 4500 mRNA transcripts. Knocking-out DHX36 increases the number of BG4 foci in cells, and DHX36 binding to G4s increases translation and prevents the accumulation of translationally repressed mRNAs (58). Chen *et al.* recently co-crystallised DHX36 bound to the c-Myc G4, providing valuable insight into the mechanisms underpinning helicase mediated G4 unwinding (55,56,78). Here, we investigated the interactions between DHX36 and Task3 mRNA through direct and targeted RNA immunoprecipitation. We show that Task3 mRNA is a functional target of DHX36, consequently relieving the translational repression elicited by the 5′ UTR G4 and increasing cellular K^+^ leak currents typical of Task3, further suggesting the maintenance of the G4 structure within a cellular context. A transcriptome-wide study utilising DHX36 KO lines found a 2log-fold reduction in Task3 mRNA expression upon DHX36 KO (58). When evaluating evidence that the half-life for GGC-rich and G4-containing mRNAs is reduced upon DHX36 knock-down (80), that stalled translational complexes are triaged to P-bodies and stress-granules (79) along with our data outlining a substantial relationship between DHX36 and Task3 translational efficiency, it is likely that a DHX36 KO leads to the degradation of the mRNA and stalled ribosomal complexes at the Task3 (GGN)_13_ repeat through ribosomal surveillance degradation pathways.

It is important to note that the activity of K^+^ leak channels is largely time and voltage independent, and that control of K^+^ leak currents through gate closing or chemical stimuli occurs at low basal levels (81). Therefore, regulating the extent of K^+^ leak currents within cells occurs during the steps preceding membrane insertion, through the control of translation, Golgi export, and membrane trafficking of K2P channels (82). Identification of a translationally repressive moiety within Task3 mRNA therefore represents an important regulatory step before Task3 membrane insertion. This alters the K^+^ conductance within cells, as shown through the resulting disinhibition by G4 deletion and mutation, as well as DHX36 mediated G4 unwinding on Task3 expression and resting membrane potential. These data therefore suggest a complex combination of both cis- and trans-acting regulatory mechanisms for membrane expression of Task3 to control K^+^ leak currents. Task3 can be expressed as a homodimer or co-expressed with its paralog Task1 as a functional heterodimer (83). Database mRNA sequences for Task1 show an identical (GGC)_4_ repeat present at the very 5′ end of the 5′ UTR, which may in fact be an incomplete and truncated sequence, potentially showing more similarity in length and composition to Task3 if fully characterised. If more extensive, it could suggest that this mechanism may not be exclusive to Task3 and that G4 control of expression is maintained within the wider Task family.

It has been proposed that G4s are also a key driver of subcellular localisation of particular mRNAs. For instance, Subramanian *et al.* found that of the identified dendritic mRNAs, ∼30% had sequences in their 3′ UTRs predicted to form G4 structures (19). Mutating these potential G4-forming sequences showed that delivery of PSD-95 and CaMKIIα mRNA to neurites of cultured primary cortical neurons was dependent on the presence of the G4-forming sequence within their 3′ UTRs (19,20,84). Until now, the majority of localization signals have been attributed to 3′ UTR motifs, however, Muslimov *et al.* showed that insertion of the FXS associated *FMR1* 5′ UTR (CGG)_24_ repeat into α-tubulin 5′ UTR induced re-localisation from the neuronal somata to dendrites of cultured rat sympathetic neurons (85). Here, we show that the endogenously present (GGN)_13_ repeat, containing two separate GGC tracts of five and four repeating units, within the 5′ UTR of Task3 mRNA is also able to facilitate neurite localisation. Upon mutation and deletion of the (GGN)_13_ G4 sequence, the distance that Task3 mRNA was detected from the cell body of cultured mouse cortical neurons was reduced by 59% and 83% respectively (Figure 4C), suggesting a dependence on the presence of the (GGN)_13_ G4 structure for Task3 mRNA localisation. Importantly, this is the first observed instance of an endogenously situated 5′ UTR G4 controlling distal localisation of an mRNA in neurons. Task3 mRNA was also detected as dense puncta along intact neurite projections when comparing the diffuse eGFP staining and intact DIC images for both Figures 4 and 5. This suggests that Task3 mRNA is likely packaged within cytoplasmic messenger ribonucleoprotein (mRNP) complexes, as is observed for distally transported mRNAs within neurons (86–88). Further, the detection of DHX36 in the axonal projections (Figure 5B, right) also suggests the presence of the correct and relevant machinery able to overcome G4-induced translational repression within the same distal compartments as Task3 mRNA.

Current models of G4 regulated local translation of neuronal mRNAs postulate that G4 mRNA is transported in a translationally repressed state by G4 binding proteins (G4-RBPs) such as FMRP. Upon neuronal activity and mGluR activation, G4-RBPs such as FMRP dissociate from their G4 containing mRNA thereby partially relieving the translational repression and allowing for spatiotemporally regulated translation of key mRNAs (67,88). For Task3, we observe both an increase in mRNA concentration and protein translation upon 50 μM glutamate treatment (Figure 5C and D), which suggests a similar mechanism of activity dependent regulation as is seen for other neuronal G4 containing mRNAs. Axonal transport is reported to occur at speeds of less than 2 μm/s (89). Therefore, for long-distance neuronal projections, the presence of translationally repressed pools of mRNA at sites of neuronal activity is key to coupling this rapid increase of protein synthesis. We hypothesise that the increase in membrane expression of Task3 leak channels is a mechanism of maintaining membrane conductance to K^+^ during sustained neuronal activity to both sustain neuronal activity as well as a mechanism of neuroprotection during prolonged activity. Therefore, a combination of readily responsive, distally localised mRNA, as well as the slower replenishment of Task3 mRNA to these distal sites would likely be key to facilitating the increase in Task3 protein expression observed in response to neuronal activity. Recent work has suggested that CGG repeats within the 5′ UTR of FMR1 mRNA are required for the mGluR activity-dependent increase in FMRP synthesis within neurons (90), opening up the possibility of a role for the (GGN)_13_ repeat in mediating increases in Task3 expression in response to neuronal activity.

Taken together, our data suggest that a (GGN)_13_ repeat within the 5′ UTR of Task3 mRNA forms a parallel G4 that is responsible for the regulation of localisation and translation of Task3 K2P leak channels. Overexpression of the G4 helicase DHX36 overcomes the translational repression elicited by the 5′ UTR G4, thereby increasing cellular K^+^ leak currents and decreasing resting membrane potentials. Task3 is a protein with a narrow window of physiological expression, with over- and under-expression implicated in cancer and neurodevelopmental disorders respectively. Given our findings, we suggest that this 5′ UTR G4 is central to the regulated expression of Task3 leak channels *in vivo*.

## DATA AVAILABILITY

5′ RACE sequencing data have been deposited in GenBank [Accession Number: MN510330].

Stellaris^®^ RNA FISH probe designer was used for Task3 mouse Quasar^®^ 570 nm design https://www.biosearchtech.com/support/tools/design-software/stellaris-probe-designer.

QGRS Mapper: a web-based server for predicting G4s in nucleotide sequences http://www.bioinformatics.ramapo.edu/QGRS/index.php.

## Supplementary Material

gkaa699_Supplemental_File

## Notes

Present address: Mark J. Coldwell, School of Biological Sciences, University of Southampton, Southampton, Hampshire SO17 1BJ, UK.
