# Supplementary material for: A 5′ UTR GGN repeat controls localisation and translation of a potassium leak channel mRNA through G-quadruplex formation
Source: Nucleic Acids Res. 2020 Sep 1;48(17):9822–39. doi: 10.1093/nar/gkaa699 (PMC7515701; doi:10.1093/nar/gkaa699)
Supplement: gkaa699_Supplemental_File [file gkaa699_supplemental_file.pdf]

Supplementary Information and Data

SUPPLEMENTARY MATERIALS AND METHODS

EVOS Brightfield Live-Cell Imaging

Brightfield images of cells in culture were taken on an EVOS XL Core Imaging System (Thermo Fisher).

Flow Cytometry

HEK293 cells were plated and transfected for the desired time course as previously described. HEK293 cells were isolated through enzymatic detachment (TrypLE, Gibco), followed by cell pelleting and washing in ice cold PBS (Gibco). For cell death analysis, propidium iodide (PI, Sigma) solution was added to samples at a final concentration of 0.1 mg/mL, incubated on ice for 15 min and cell solutions then analysed on the Merck Guava® easyCyte™ benchtop system. Briefly, control non-PI stained cells were used to correctly set up the detection and gating system for forward scatter, side scatter and an appropriate laser for the propidium iodide to ensure all cell populations were captured during the analysis. 10^4 cells were analysed per sample. Data was analysed using the Guava® InCyte software.

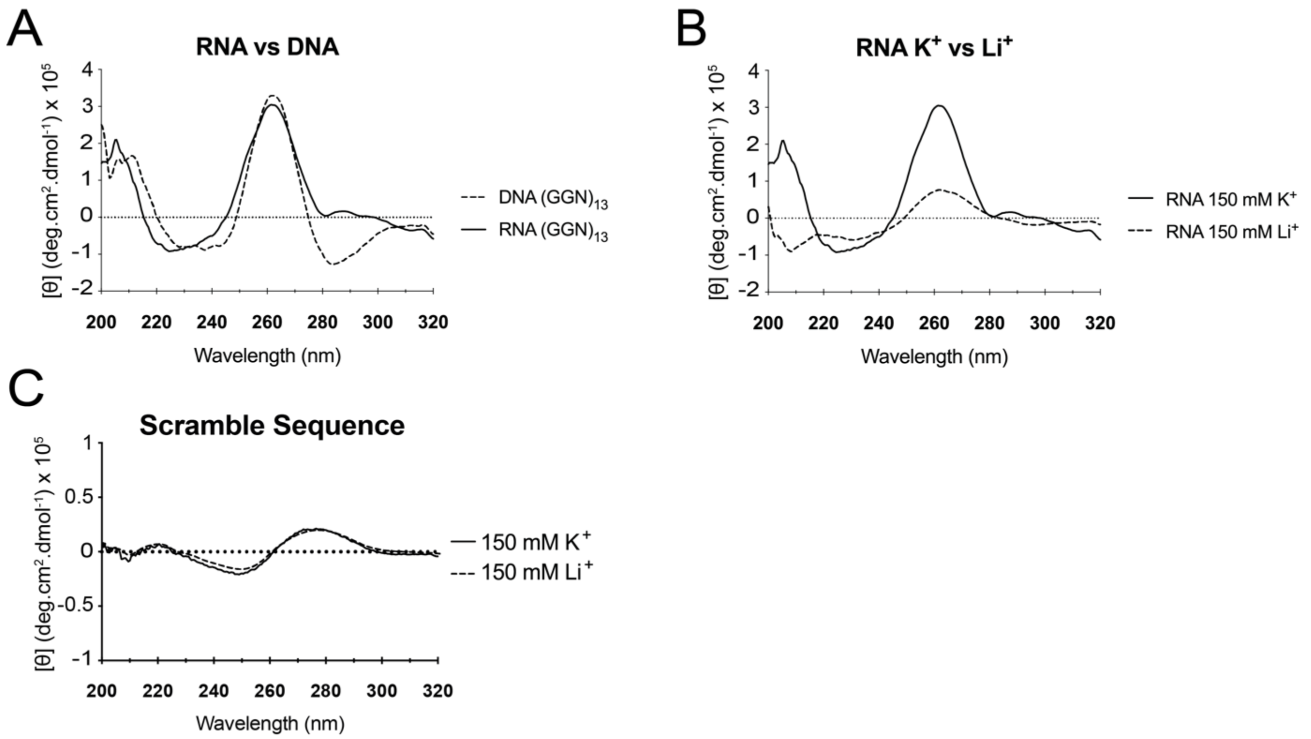

Figure S1 – Supplementary characterisation of oligonucleotides through circular dichroism – (**A**) The CD spectra of 10 μM Task3 WT $(GGN)_{13}$ RNA oligonucleotides (Black solid) or DNA oligonucleotides (Black dashed) folded in potassium phosphate buffer supplemented with KCl to 150 mM K^+. (**B**) The CD spectra for 10 μM Task3 $(GGN)_{13}$ RNA oligonucleotides folded in potassium phosphate buffer supplemented with KCl to 5 or 150 mM K^+ or folded in sodium phosphate buffer supplemented with LiCl to 150 mM Li^+. (**C**) CD spectra of a 10 μM GC-rich length-matched scramble oligonucleotide sequence folded in potassium or sodium phosphate buffer supplemented with KCl or LiCl to 150 mM K^+/Li^+. CD spectra were obtained from five repeated acquisitions to increase the signal:noise ratio with a representative trace shown.

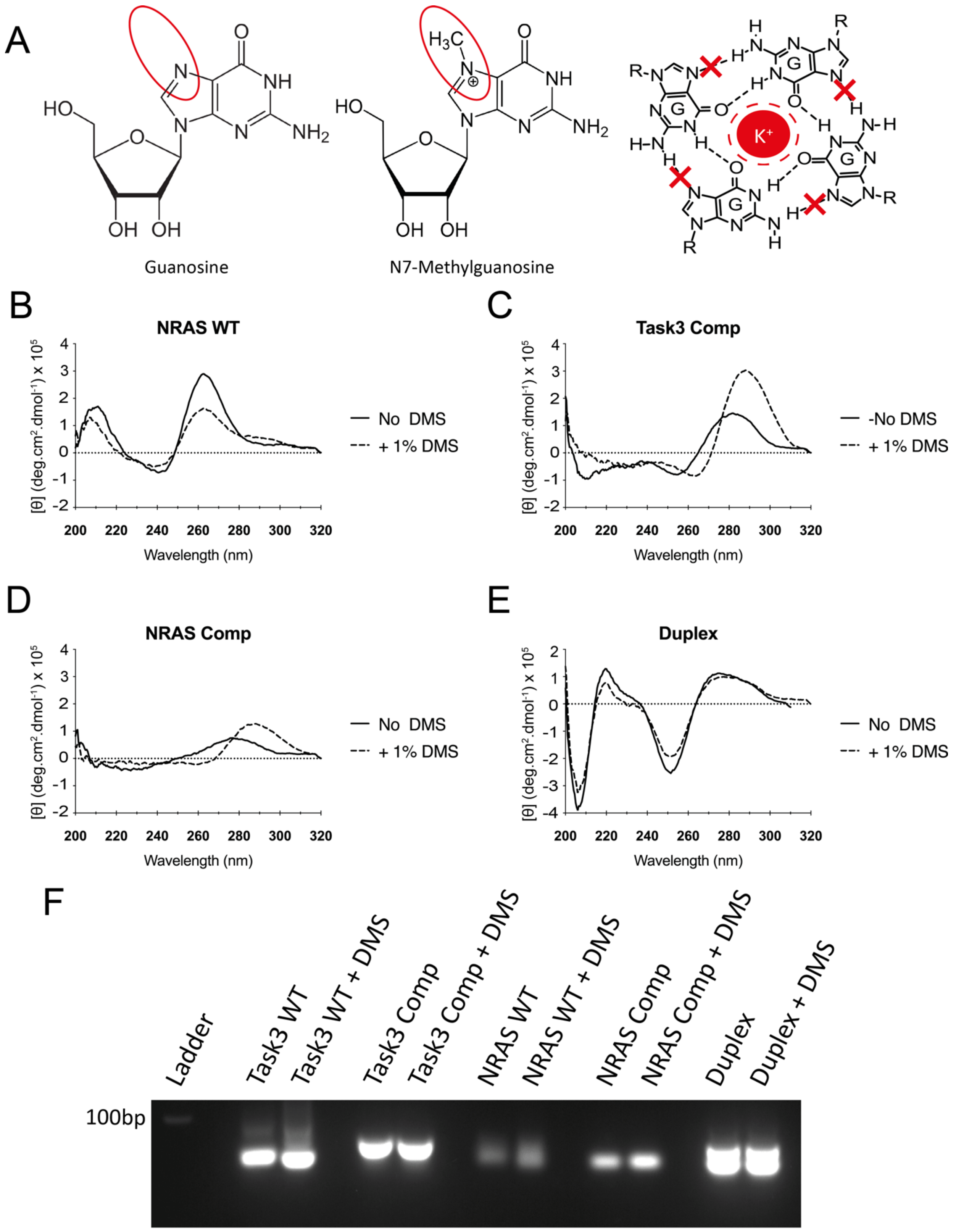

Figure S2 – 1% DMS treatment inhibits G4 formation for Task3 WT (GGN)$_{13}$ and NRAS WT G4 – (**A**) Schematic showing the basis of DMS induced N7-methylation of guanine, inhibiting the Hoogsteen hydrogen bonding underpinning G-quartet formation. (**B**) CD spectra for NRAS WT G4 at 150 mM K^+ either in the presence of absence of 1% DMS pre-folding. (**C**) CD spectra for Task3 Comp (CCN)$_{13}$ at 150 mM K^+ either in the presence of absence of 1% DMS pre-folding. (**D**) CD spectra for NRAS Comp at 150 mM K^+ either in the presence of absence of 1% DMS pre-folding. (**E**) CD spectra for the duplex control at 150 mM K^+ either in the presence of absence of 1% DMS pre-folding. (**F**) All aforementioned samples run on 1% agarose gel post-CD analysis. CD spectra were obtained from five repeated acquisitions to increase the signal:noise ratio with a representative trace shown.

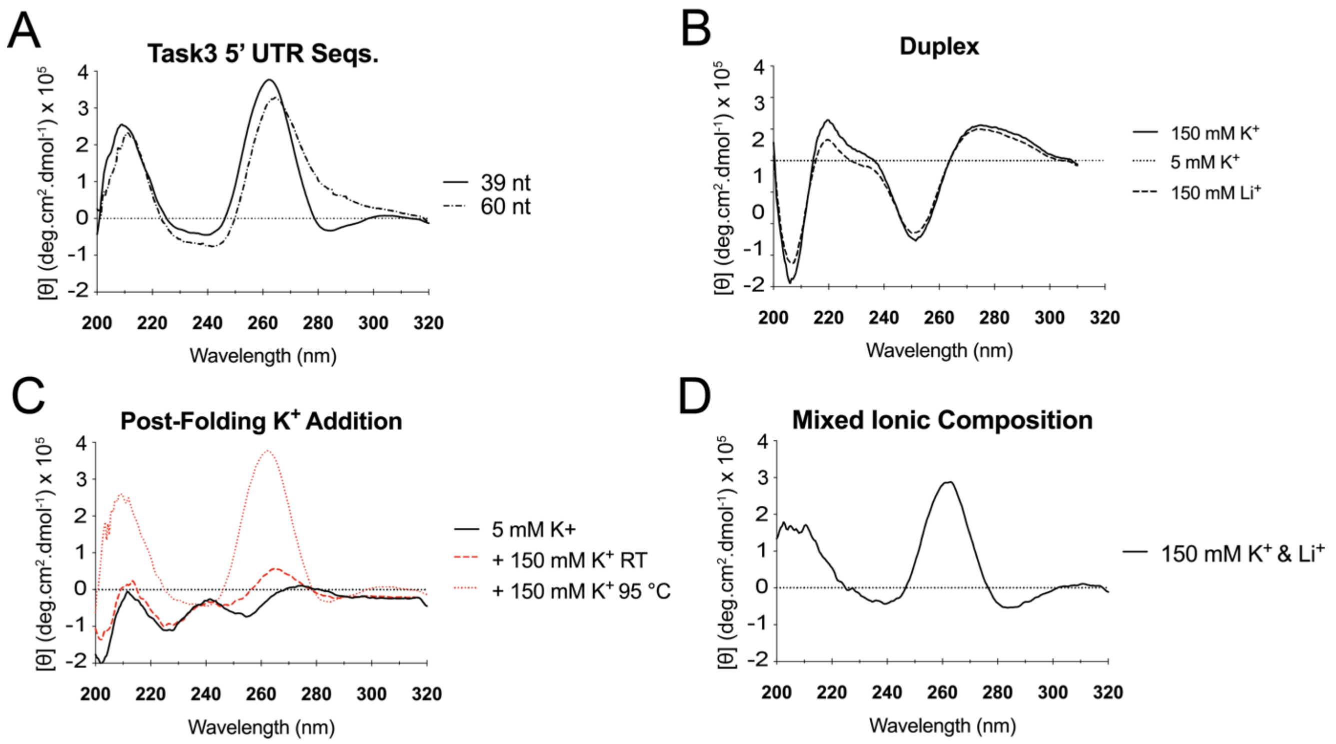

Figure S3 – Supplementary characterisation of oligonucleotides through circular dichroism – (**A**) CD spectra of 10 µM oligos containing either the Task3 WT $(GGN)_{13}$ repeat or a longer 60 nt sequence containing the WT $(GGN)_{13}$ repeat with the 11 nt upstream and 10 nt downstream found within the 5' UTR, folded in potassium phosphate buffer supplemented with KCl to 150 mM K^+. (**B**) The CD spectra for 10 µM duplex forming oligos folded in potassium phosphate buffer supplemented with KCl to 5 or 150 mM K^+ or folded in sodium phosphate buffer supplemented with LiCl to 150 mM Li^+. (**C**) The CD spectra of 10 µM Task3 WT $(GGN)_{13}$ folded in potassium phosphate buffer supplemented with KCl to 5 mM K^+ (Black). The sample was subsequently supplemented with KCl to 150 mM K^+ and incubated for 30 mins before spectral acquisition (Red dashed) followed by denaturation at 95 °C and cooling before spectral acquisition (Red dotted). (**D**) The CD spectra of 10 µM Task3 WT $(GGN)_{13}$ folded in sodium phosphate buffer supplemented with KCl to 150 mM K^+ and LiCl to 150 mM Li^+. CD spectra were obtained from five repeated acquisitions to increase the signal:noise ratio with a representative trace shown.

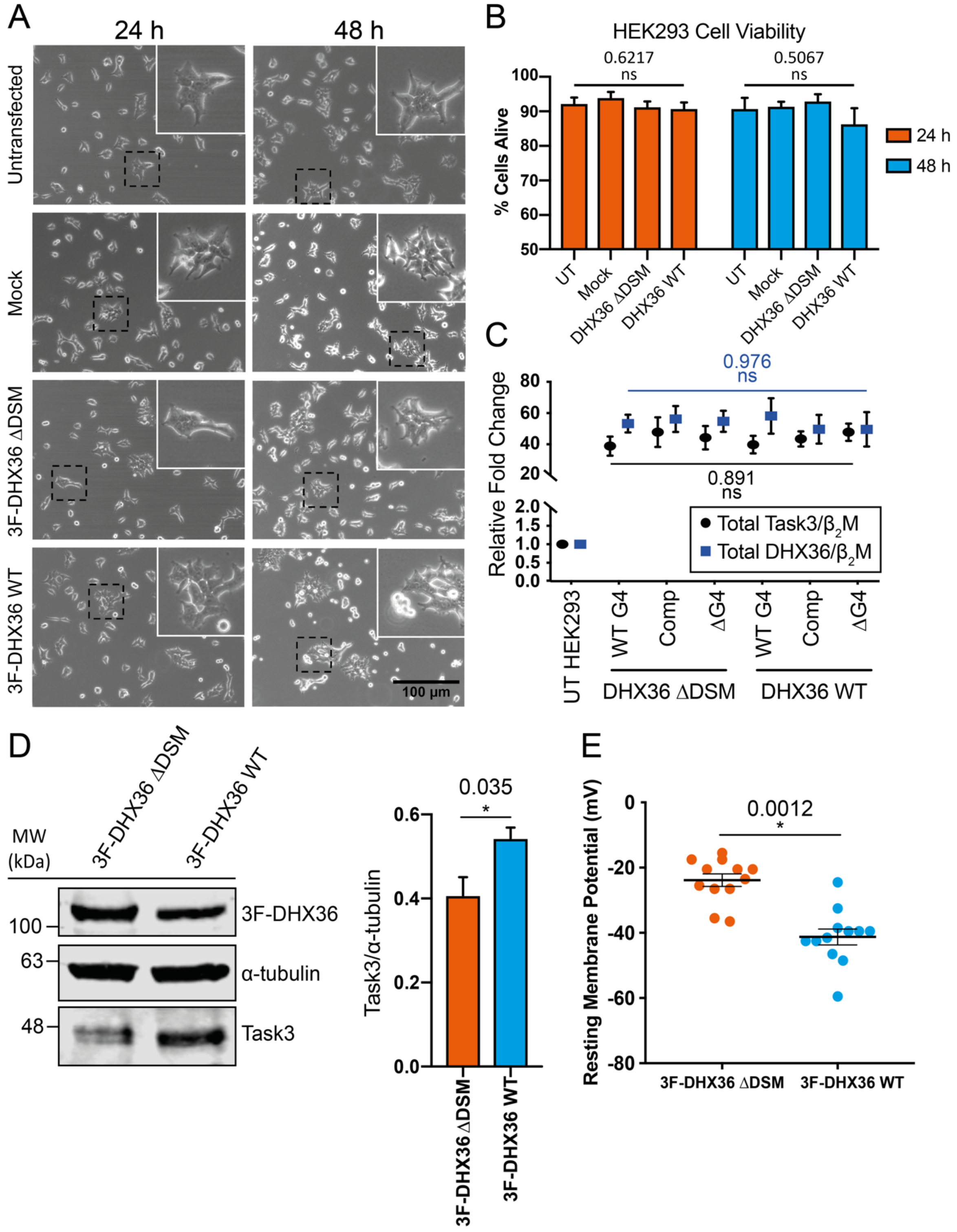

Figure S4 – Supplementary characterisation of the effects of DHX36 overexpression on cell viability and Task3 expression – (**A**) EVOS live-cell brightfield images of HEK293 cells transfected for either 24 or 48 h with 3F-DHX36 WT and 3F-DHX36 ΔDSM compared to transfected and mock-transfected controls. (**B**) Percentage cell viability for each transfection condition measured through live/dead counting of propidium iodide fluorescence. (**C**) Total Task3 and DHX36 mRNA quantified in each co-transfection condition to signify the relative increase in abundance for Task3 and DHX36 respectively when overexpressed. Each condition is normalised such that their untransfected sample = 1. (**D**) Western blotting of HEK293 lysates transfected with 3F-DHX36 ΔDSM or 3F-DHX36 WT (Left) and quantified for endogenous Task3 expression (Right). (**E**) Whole cell patch clamp recordings of HEK293 cells transfected with Fluc-3F or 3F-DHX36. N=3(**B**) or 4(**C** and **E**) biological replicates where error bars represent SEM. Statistical analyses were carried out by one-way ANOVA (**B** - 24 h: $F(3, 12) = 0.8453$, $p=0.6127$, 48 h: $F(3, 12) = 0.8453$, $p=0.5067$. **C** – Task3 (Black): $F(5, 20) = 0.3216$, $p=0.891$, DHX36 (Blue): $F(5, 20) = 0.1509$, $p=0.976$), or by unpaired two-tailed T-tests: **D** (Right) and **E**, p values shown.

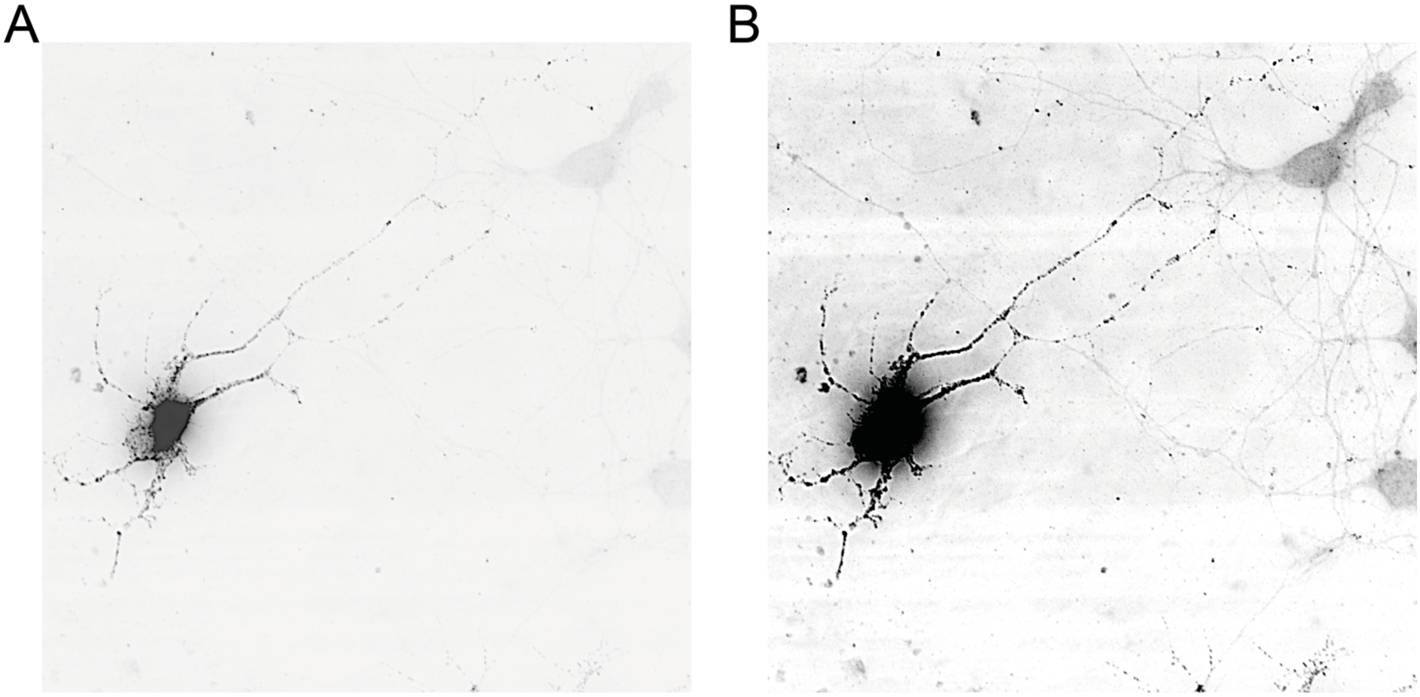

Figure S5 – RNA FISH of Task3^{M1}-eGFP in DIV10 primary cortical neurons at a short exposure to define mRNA granules within the cell body (**A**), and at a long exposure to define mRNA granules in the neurite projections (**B**).

Primer	↔	5'-3'
FLAG	F	ACTACAAAGACCATGACGGTG
	R	TCGTCAGCCTTGTAGTCGATGTC
β2M	F	TTCATCCATCCGACATTGAAG
	R	ATCCAATCCAAATGCGGC
NPTII	F	CTCACCTTGCTCCTGCCGAGA
	R	CGCCTTGAGCCTGGCGAACAG
NRAS Mus	F	CAAGGACAGTTGACACAAAGC
	R	TGTCTTACTACATCAGCACACAG
Task3 Mus	F	CACAACTATCGGATATGGAC
	R	CTCCATAGAAACTTCAGTGTTG
H1F0 Mus	F	CCATCGGAAGGCTCCTTGAA
	R	CACTAGGCGCTTGATGGACA
β-Actin Mus	F	CAGCCTTCCTTCTTGGGTATG
	R	AGCTCAGTAACAGTCCGCCT
GAPDH Mus	F	TGAACGGGAAGCTCACTGG
	R	TCCACCACCCTGTTGCTGTA
NRAS Hum	F	GAAACCTCAGCCAAGACCAGAC
	R	GGCAATCCCATACAACCCTGAG
Task3 Hum	F	TACTTTGCGATCACGGTCATC
	R	GTAGCGCACGAAGGTGTTC
H1F0 Hum	F	CTGGCTGCCACGCCCAAGAA
	R	CGGCCCTCTTGGCACTGGAC
DHX36 Hum	F	GGGTCATGGAGGTAACCGAG
	R	CTCTCCGCTTCCTTGTTCTTC
DHX36 ΔDSM	F	ATGTGGTACGCAAAAAACAGGGGCAGAAGAACAAGG
	R	CCGGCCCCTGCCGCCTCG
DHX36 E335A	F	CGTACTTGATGCAATCCATCCATGAAAG
	R	ATATGACTAACACTGGAC
Task3 RACE GSP	/	AACGTGCGGACTCTGTCC

Figure S6 – List of primers used for PCR and qPCR obtained from Sigma.
